# Motor protein Kif6 regulates cilia motility and polarity in brain ependymal cells

**DOI:** 10.1242/dmm.050137

**Published:** 2024-02-29

**Authors:** Maki Takagishi, Yang Yue, Ryan S. Gray, Kristen J. Verhey, John B. Wallingford

**Affiliations:** ^1^ Department of Molecular Biosciences, Patterson Labs, The University of Texas at Austin, TX 78712, USA; ^2^ Department of Cell and Developmental Biology, University of Michigan Medical School, Ann Arbor, MI 48109, USA; ^3^ Departments of Nutrition and Pediatrics, Dell Pediatric Research Institute, The University of Texas at Austin, Dell Medical School, Austin, TX 78712, USA

**Keywords:** Kinesin, Multiple motile cilia, Ependymal cell cilia beating, Cilia, Kif6

## Abstract

Motile cilia on ependymal cells that line brain ventricular walls beat in concert to generate a flow of laminar cerebrospinal fluid (CSF). Dyneins and kinesins are ATPase microtubule motor proteins that promote the rhythmic beating of cilia axonemes. Despite common consensus about the importance of axonemal dynein motor proteins, little is known about how kinesin motors contribute to cilia motility. Here, we show that Kif6 is a slow processive motor (12.2±2.0 nm/s) on microtubules *in vitro* and localizes to both the apical cytoplasm and the axoneme in ependymal cells, although it does not display processive movement *in vivo*. Using a mouse mutant that models a human Kif6 mutation in a proband displaying macrocephaly, hypotonia and seizures, we found that loss of Kif6 function causes decreased ependymal cilia motility and, subsequently, decreases fluid flow on the surface of brain ventricular walls. Disruption of Kif6 also disrupts orientation of cilia, formation of robust apical actin networks and stabilization of basal bodies at the apical surface. This suggests a role for the Kif6 motor protein in the maintenance of ciliary homeostasis within ependymal cells.

## Teaser

**Summary:** Kif6 localizes to the axonemes of ependymal cells. *In vitro* analysis showed that Kif6 moves along microtubules and that, in mouse, its loss decreases cilia motility and cilia-driven flow, resulting in hydrocephalus.

## INTRODUCTION

Multiciliated cells (MCCs) have dozens of motile cilia that are planar polarized and coordinately beat to generate a directional flow across the epithelium (Brooks and Wallingford, 2014). Cilia-driven directional fluid flow maintains mucus clearance in the respiratory tract, transport of gametes in the reproductive system and circulation of cerebrospinal fluid (CSF) in the brain (Spassky and Meunier, 2017); however, recent studies have revealed subtle but important differences in these MCC populations (Konjikusic et al., 2018; Roberson et al., 2020).

Dyneins and kinesins are ATPase microtubule motor proteins that drive diverse motion in cilia and flagella (Gennerich and Vale, 2009). For example, axonemal dynein complexes, such as outer and inner dynein arms, are arrayed along microtubule doublets to generate a motive force for cilia beating (Ishikawa, 2017). However, while both kinesins and dyneins help facilitate intraflagellar transport of cargoes necessary to build and maintain cilia (Prevo et al., 2017), the function of several ciliary kinesins remains poorly defined (Konjikusic et al., 2021).

The kinesin-9 family, for example, comprises two subfamilies – kinesin-9A and kinesin-9B (Wickstead et al., 2010) – that have been implicated in cilia function but remain only cursorily studied. In *Trypanosome brucei,* both family members, KIF9A and KIF9B, are localized to the axoneme and their knockdown reduced flagellum motility (Demonchy et al., 2009). The KIF9A homologues KLP1 in *Chlamydomonas reinhardtii* and Kif9 in vertebrates have recently been shown to be active motor proteins that act in the central pair to facilitate ciliary beating (Konjikusic et al., 2023; Han et al., 2022).

Not much is known about members of the kinesin-9B subfamily. The kinesin-like protein Kif6 in vertebrates has been implicated in cardiovascular disease in humans (Ruiz-Ramos et al., 2015), although a meta-analysis of multiple case-control studies failed to find evidence of this association (Assimes et al., 2010). Instead, more-recent studies in fish, mouse and human did suggest a role for Kif6 in cilia in the brain (Gray et al., 2021; Konjikusic et al., 2018). In both zebrafish and mouse, loss of Kif6 function is associated with hydrocephalus and a decrease in the number of cilia within ventricles of the adult brain (Gray et al., 2021; Konjikusic et al., 2018); however, little else of Kif6 function regarding cell biology is known.

Here, we provide evidence that Kif6 functions as a canonical plus-end directed kinesin motor capable of slow, processive, microtubule-based movement *in vitro*. In mouse, loss of Kif6 function elicits a complex array of cilium-related phenotypes in the brain, including defects of cilia beating, CSF flow and planar polarization of basal bodies. Over time, ependymal cilia in Kif6 mutant mouse are lost, possibly due to defects in apical actin assembly and subsequent destabilization of basal bodies. Thus, our data suggest that Kif6 plays several roles in ependymal cell cilia homeostasis and neurodevelopment in mammals, and might shed light on Kif6-related human pathologies.

## RESULTS

### Kif6 is an active motor protein that ‘walks’ towards microtubule plus ends

We previously reported a macrocephaly patient with a mutation in *KIF6* that eliminates part of the coiled-coil stalk region and the C-terminal tail domain (Konjikusic et al., 2018), i.e. the putative cargo-binding domain (Piddini et al., 2001). We also generated mice with a similar mutation eliminating the C-terminal stalk and tail domains of Kif6, which lead to variably penetrant hydrocephalus and a reduction in the number of ependymal cell cilia (Konjikusic et al., 2018). Since the motor activity of Kif6 has never been determined, these findings led us to perform *in vitro* studies.

**Fig. 1.**
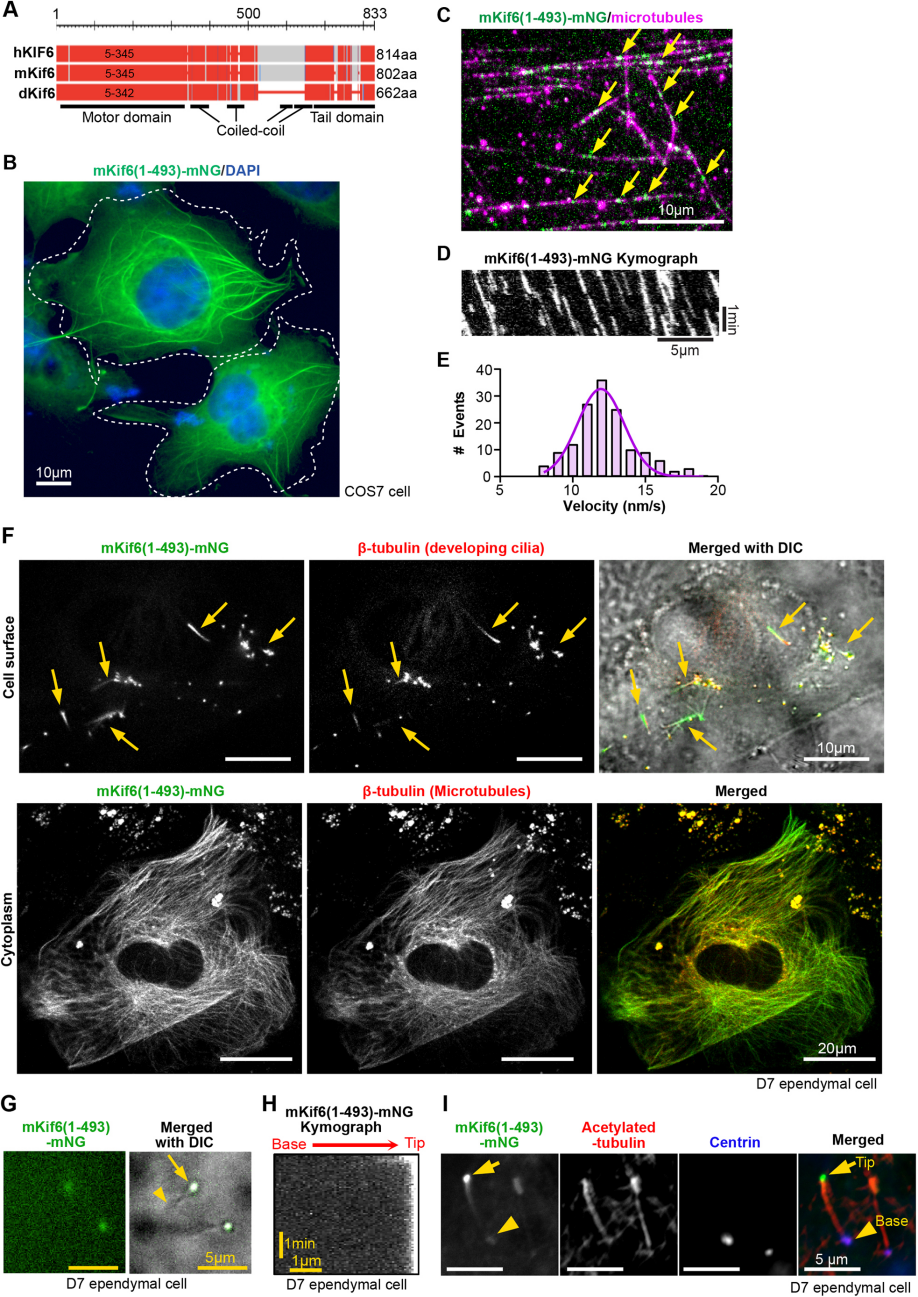
**Kif6 is an active motor that localizes to cytoplasmic and ciliary microtubules.** (A) Schematic representation of human, mouse and zebrafish Kif6 proteins (hKIF6, mKif6 and dKif6, respectively). The predicted motor domain is the region of amino acids (aa) 5-345 within hKIF6, 5-345 aa within mKif6 and 5-342 within dKif6. Red regions are highly conserved positions within human, mouse and zebrafish proteins. (B) Representative image of mNeonGreen (mNG)-tagged mKif6(1-493) expressed in COS-7 cells. White dashed lines indicate the outlines of transfected cells. Nuclei were stained with DAPI (blue). (C) Representative still image from single-molecule motility assay of mKif6(1-493)-mNG molecules (green) on taxol-stabilized microtubules (magenta). Arrows indicate individual mKif6(1-493)-mNG molecules on microtubules. (D) Kymograph of mKif6(1-493)-mNG movement from the single-molecule motility assay shown in C. Time is on the *y*-axis and distance is on the *x*-axis. (E) The velocities of individual mKif6(1-493)-mNG were determined from kymographs and plotted as a histogram for the population. The curve was fit with a normal distribution. Velocity is described as mean±s.d. motility events (*n*=143) analyzed across three independent experiments. (F) Images of a cultured developing ependymal cell at D7 expressing mKif6(1-493)-mNG. Top panels: immunostaining for mKif6(1-493)-mNG (green) and -β-tubulin (red) in black and white (left and middle); merged colored image (right) with digital image-correlation (DIC) on cell surface to see cilia (indicated by yellow arrows). Bottom panels: immunostaining for mKif6(1-493)-mNG and β-tubulin (red) in black and white (left and middle); merged colored image (right) of the same cell at the cytoplasmic plane. (G) Still frame from live imaging of mKif6(1-493)-mNG in a developing short cilium of a D7 ependymal cell. Arrow indicates a ciliary tip; arrowhead indicates a ciliary base. See Movie 2. (H) Kymograph of mKif6(1-493)-mNG movement along cilia in the live imaging shown in G. (I) Ependymal cells expressing mKif6(1-493)-mNG immunostained against acetylated-tubulin (red) to visualize cilia and against centrin (magenta) to visualize the ciliary base. Arrow indicates a ciliary tip; arrowhead indicates a ciliary base.

The alignment of the protein sequences of human (h), mouse (m), and *Danio rerio* (d) Kif6 proteins showed that the overall domain organization is highly conserved (Fig. 1A, Fig. S1A,B). When overexpressed in COS-7 cells, full-length (FL) mouse Kif6-EGFP (hereafter Kif6 FL-EGFP) did not localize to microtubules but, rather, showed a diffuse localization within the cytosol (Fig. S1C). In *in vitro* single-molecule motility assays, Kif6 FL-EGFP did not bind to taxol-stabilized microtubules (Fig. S1D), suggesting that full-length mKif6 is regulated by autoinhibition, which is common for kinesin proteins (Verhey and Hammond, 2009). To examine the motility properties of mKif6, we generated a truncated version of mKif6 (1-493) tagged to mNeonGreen [hereafter referred to as mKif6(1-493)-mNG], which includes the motor domain and the first two coiled-coil segments for homodimerization (Fig. 1A). When overexpressed in COS-7 cells, mKif6(1-493)-mNG was found both along microtubules and diffusely in the cytoplasm (Fig. 1B).

To visualize Kif6 movement directly, we carried out *in vitro* single-molecule motility assays and found that mKif6(1-493)-mNG moved processively along microtubules with a speed of 12.2±2 nm/s (mean±s.d.) (Fig. 1C–E, Movie 1). Similar speed (15.5±4.4 nm/s) was obtained with a truncated version of dKif6 (not shown). This slow rate of processive movement along microtubule tracks is similar to that reported previously for the related kinesin Kif9 (Konjikusic et al., 2023).

To test whether Kif6 protein moves processively towards the plus or the minus end of microtubules, we repeated the single-molecule motility assays in the presence of the kinesin-3 protein KIF1A, as a truncated version of this motor protein – KIF1A(1-393) that lacks amino acids (aa) 1–393 – is known to move with high speed and processivity towards the plus ends of microtubules (Hammond et al., 2009; Soppina et al., 2014). For these experiments, we used KIF1A(1-393)-Halo^JFX554^, for which KIF1A(1-393) had been conjugated to the HaloTag and labeled with the HaloTag ligand JFX554. Given the differences in speed between the kinesin-3 KIF1A and the kinesin-6 proteins hKif6, mKif6 and dKif6, we first imaged motility of KIF1A along microtubules by using a rapid frame rate (ten frames per second) and then the motility of the KIF6 proteins along the same microtubule by using a slower frame rate (one frame every 3 s). Kymographs revealed that both mouse KIF6(1-493)-mNG (Fig. S2) and zebrafish KIF6(1-501)-mNG (not shown) moved in the same direction as KIF1A, demonstrating that they move processively towards the microtubule plus end.

### Kif6 localizes to the cytoplasm and cilia of multiciliated ependymal cells

We then expressed mKif6(1-493)-mNG in developing ependymal cells, where it colocalized to β-tubulin not only in cytoplasmic microtubules but also in cilia (Fig. 1F,I). While mKif6(1-493)-mNG accumulated at the tip of cilia (Fig. 1G,I), we were unable to observe any clear movement of mKif6(1-493)-mNG within these cilia (Fig. 1H, Movie 2). Together, these data suggest that, although the Kif6 motor domain derived from either zebrafish or mouse is able to bind to and move processively along microtubules, it cannot do so appreciably inside cilia; similar behavior has been shown for other KIF9 family kinesins, such as *Chlamydomonas* Klp1 and *Xenopus* Kif9 (Konjikusic et al., 2023; Han et al., 2022).

**Fig. 2.**
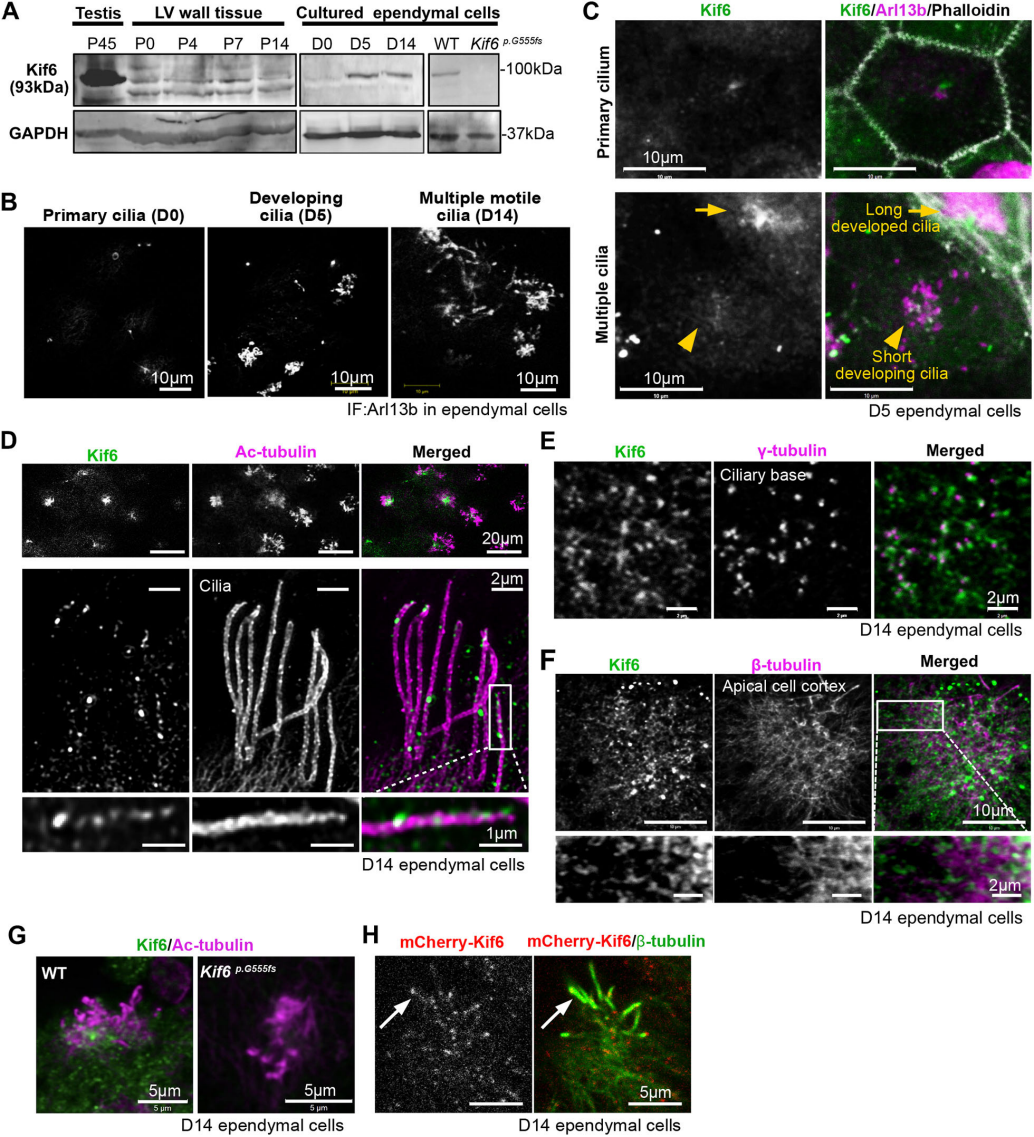
**Kif6 localizes to the cytoplasm and cilia of multiciliated ependymal cells.** (A) Western blot analysis with anti-Kif6 or anti-GAPDH in P45 mouse testis tissue, P0, 4, 7, or 14 mouse LV wall tissues, or D0, 5, or 14 cultured ependymal cell lysates. Right panels show D14 ependymal cell lysate from WT or *Kif6^p.G555fs^* mice. (B) Representative image of cilia formation in cultured ependymal cells at D0, D5 or D14. Ependymal cells were immunostained with anti-Arl13b. (C) Immunostaining with anti-Kif6 (green), anti-Arl13b (magenta, as a cilium marker) and phalloidin (gray, as a cell border marker) in differentiating ependymal cells at D5. Upper panels show a primary ciliated precursor cell. Lower panels show multiple ciliated differentiating ependymal cells. (D) Super-resolution image of differentiated ependymal cell cilia at D14 immunostained with anti-Kif6 (green) and anti-acetylated (Ac)-tubulin (magenta). Top panels show Kif6 expression in the differentiated ependymal cells at low magnification. Middle panels show representative ependymal cilia in one ependymal cell. Boxed area is enlarged in the bottom panels. (E) Immunostaining with anti-Kif6 (green) and anti-γ-tubulin (magenta, as a basal foot marker) at the cytoplasmic ciliary base. (F) Immunostaining with anti-Kif6 (green) and anti-β-tubulin (magenta, as a microtubules marker) at the apical cell cortex. (G) Immunostaining with anti-Kif6 (green) at cilia and ciliary base in D14 ependymal cell from WT or *Kif6^p.G555fs^* as negative control. (H) mCherry-Kif6 (white in left panel, red in right panel) expressing cells are immunostained with anti-β-tubulin (green). Arrow indicates mCherry-Kif6 localization at cilia.

We next set out to define endogenous Kif6 protein localization using immunochemistry. Antibody specificity was confirmed using western blot analysis of primary ependymal cell lysates derived from WT and *Kif6^pG555fs/pG555fs^* homozygous mutant (hereafter referred to as *Kif6^pG555fs^*) mice. The *Kif6^pG555fs^* mutation was predicted to truncate the Kif6 protein, thus removing the antigen region recognized by anti-Kif6 antibody. Western blotting confirmed a single band of Kif6 protein expressed in testis and developing short cilia and developed long cilia (Fig. 2A). Interestingly, we consistently observed multiple bands when using anti-Kif6 antibody in lysates derived from lateral ventricular (LV) wall tissue. Because LV wall tissue contains many cells other than pure populations of primary cultured ependymal cells, robust Kif6 expression might be masked in these lysates. Next, we enriched for ependyma from primary LV wall tissue and confirmed ependymal cell differentiation at day (D)0, D5, and D14. At D0, the precursor cells had a short Arl13b-positive cilium (primary cilia). At D5, the cultured dish included precursor cells and immature ependymal cells that had multiple short or elongating cilia. At D14, mature ependymal cells had multiple long cilia (Fig. 2B). We first observed Kif6 at D5 of differentiation, which is concomitant with the onset of MCC differentiation within cultured ependymal cells.

We next examined Kif6 localization in ependymal cells by immunostaining *in vivo*. At D5 we observed a Kif6 punctum close to a primary cilium, with increased accumulation at the base of both developing short cilia and developed long cilia (Fig. 2C). To resolve this accumulation of Kif6 in cilia we used super-resolution imaging of ependymal cell cultures at D14, showing puncta along the length of ciliary axonemes (Fig. 2D). We also observed Kif6 puncta at the apical cell cortex localized to the basal foot (BF) marker γ-tubulin (Fig. 2E) and partially colocalized to microtubules (β-tubulin) at apical cell cortex around the ciliary base, as reflected by the staining for γ-tubulin (Fig. 2F). The specificity of immunostaining with anti-Kif6 antibody was confirmed by the loss of signal at multicilia in D14 ependymal cells derived from *Kif6^pG555fs^* mutant mice (Fig. 2G). Finally, exogenously expressed mKif6 tagged to mCherry at its N-terminal end (mCherry-Kif6) localized to multicilia and the apical cell cortex together with β-tubulin (Fig. 2H). These results suggest that endogenous Kif6 localizes to the apical tubulin cortex at the base of cilia and to within ciliary axonemes.

### Kif6 regulates cilia motility in ependymal cells

**Fig. 3.**
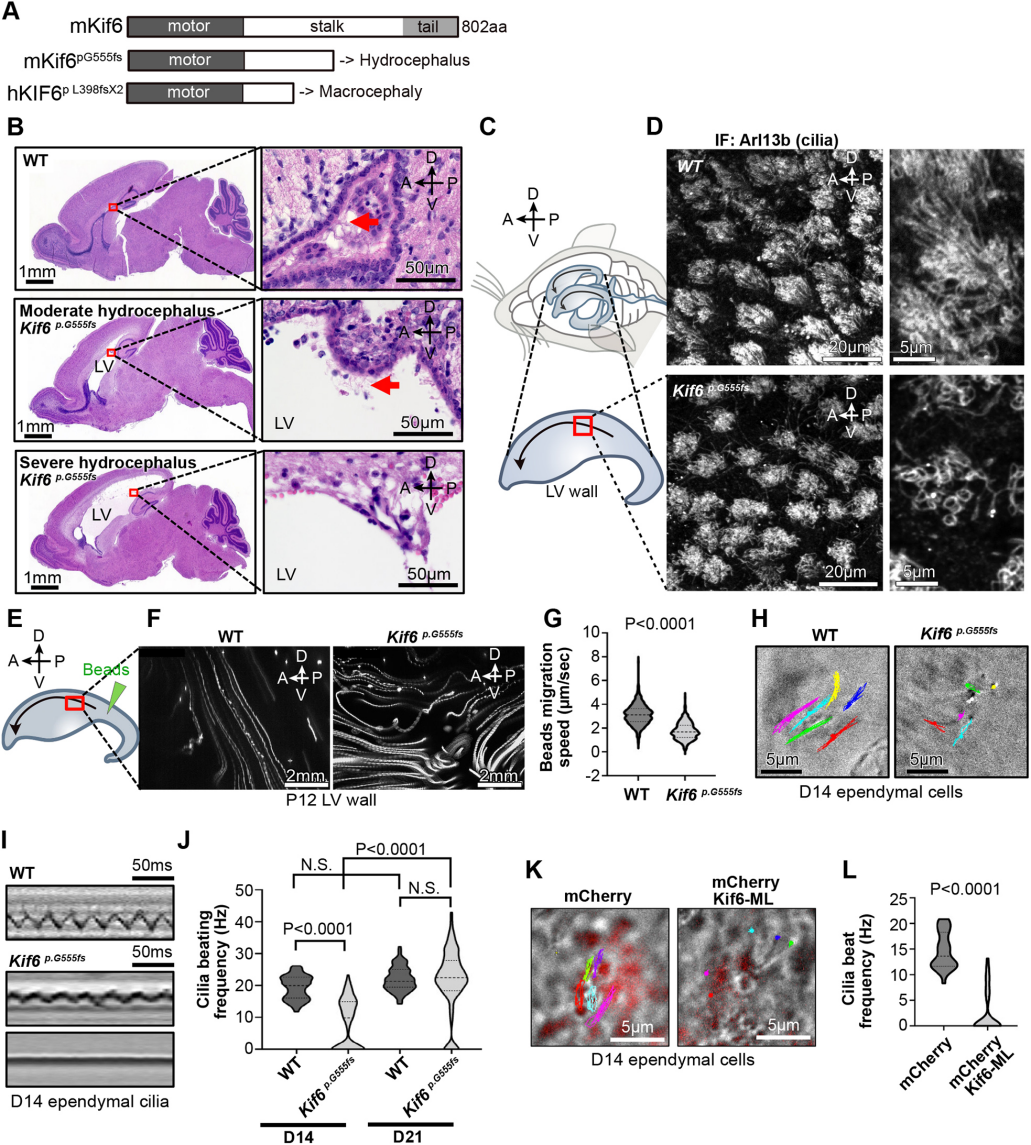
**Kif6 regulates cilia motility in mouse ependymal cells.** (A) Schematic representation of Kif6 proteins, i.e. WT mouse Kif6 (mKif6), mutant mouse Kif6^p.G555fs^ (mKif6^p.G555fs^) resulting in hydrocephalus in mice, and mutant human KIF6 (KIF6^p.L398fsX2^) causing macrocephaly in humans. (B) Images of H&E-stained sagittal sections of WT and *Kif6^p.G555fs^* mouse brains at P14. Areas boxed in red indicate the wall of lateral ventricles (LVs) and are shown magnified in the panels on the right. Arrows indicate ependymal cilia. The LV wall tissue in *Kif6^p.G555fs^* mice with severe hydrocephalus is disrupted, showing lose ependymal cells. (C) Illustration of mouse brain ventricles and dissected LV wall (top). Direction of the cerebrospinal fluid (CSF) flow is indicated by bend arrows. CSF is produced at specialized tissue – the choroid plexus – in the posterior region within LVs, and outflows through the foramen of Monro at the anterior–ventral region of the LVs, towards the third ventricle. The enlarged illustration below represents the surface of a dissected right distal LV wall. Plane-of-section compass (top left within top left image): A, anterior; P, posterior; D, dorsal; V, ventral. (D) Fluorescence microscopy images of LV wall tissue at the position indicated by the red boxed area in C show the surface cilia formation in WT or *Kif6^p.G555fs^* mice at P14. LV wall tissues were whole-mount immunostained with anti-Arl13b as a cilium marker. (E) Schematic of bead migration assay on the surface of LV wall tissue at P12. Placement of fluorescent microbeads is indicated by the green arrowhead, with their migration towards the anterior–ventral side, the direction of the CSF flow (bend arrow); see plane-of-section compass (top left). (F) Time projections of bead migration on WT or *Kif6^p.G555fs^* mouse LV wall tissue at the position indicated by the red boxed area in E; overlayed were 100 movie frames at ten frames per second (see Movies 3 and 4). (G) Bead migration speed quantified in LV walls of WT or *Kif6^p.G555fs^* mice at P12. The violin plot shows the distribution of bead migration speed with median and quartile ranges in WT (median=3.11, *n*=517 frames of 53 beads on three LV walls) and *Kif6^p.G555fs^* (median=1.67, *n*=513 frames of 58 beads on three LV walls) mice. *P*-value was determined with the Mann–Whitney test. (H) Movement tracks of cilia for 0.3 s in cultured ependymal cells from WT or *Kif6^p.G555fs^* mice at D14 (see Movies 5-7). (I) Representative kymographs of cilia in cultured WT (top panel) or *Kif6^p.G555fs^* (middle and bottom panels) mouse ependymal cells at D14. *Kif6^p.G555fs^* cilia have slow (middle panel) or no motility (bottom panel). (J) Graph represents cilia beating frequency (Hz) in cultured WT or *Kif6^p.G555fs^* mouse ependymal cells at D14. Truncated violin plot shows distribution of cilia beating frequency with median and quartile ranges in WT (median=19.9, *n*=118 cilia from three experiments) and *Kif6^p.G555fs^* (median=9.79, *n*=118 cilia from three experiments) mice at D14, and WT (median=20.82, *n*=98 cilia from two experiments) and *Kif6^p.G555fs^* (median=23.08, *n*=98 cilia from two experiments) mice at D21. *P*-values were determined with the Mann–Whitney test. (K) Movement tracks of cilia for 0.23 s in cultured mouse ependymal cell at D14, transfected with mCherry only (control) or motor-less Kif6 tagged to mCherry (mCherry-Kif6 ML) as a dominant-negative form of Kif6 (see Movies 8 and 9). (L) Graph indicates cilia-beating frequency (Hz) in mCherry- or mCherry-Kif6 ML-expressing mouse ependymal cells. Truncated violin plot shows distribution of cilia beating frequency with median and quartile ranges in mice expressing mCherry (median=13.62, *n*=32 cilia from three experiments) or mCherry-Kif6 ML (median=0.000, *n*=32 cilia from three experiments). *P*-value was determined with the Mann–Whitney test.

As noted, *Kif6^pG555fs^* mice display variably penetrant hydrocephalus (Konjikusic et al., 2018) (Fig. 3A). We observed moderate hydrocephalus with enlarged LV wall tissue in 69.2% (nine out of 13) mice, while severe hydrocephalus, often associated with a disrupted LV wall tissue and the absence of ependymal cells, was observed in the remainder (Fig. 3B). Because ependymal cilia were present in the middle part of LV wall tissue in *Kif6^pG555fs^* mice with moderate hydrocephalus (Fig. 3C,D; Fig. S3), we chose these for a more detailed analysis of ciliary phenotypes.

As ependymal cilia beating contributes to directional flow and CSF circulation in the mouse brain, we first observed the cilia-driven flow on the surface of LV wall tissue (Fig. 3E). Microbeads were placed on the surface of LV wall tissue at the middle part and flowed in a linear path along the anterior–dorsal region in wild-type (WT) LV wall tissue (Fig. 3E,F). However, bead displacement on *Kif6^pG555fs^* ventricles was significantly slower than on those of WT (Fig. 3G) and more tortuous (Fig. 3F; Movies 3 and 4).

Because knockdown of *Trypanosome* Kif9B (a homolog of Kif6) in *T. brucei* causes motility defects in the flagellum (Demonchy et al., 2009), we next asked if the *Kif6^pG555fs^* mutation also impairs ependymal cilia beating. To avoid potentially confounding effects of hydrocephalus and other environmental factors within brain ventricles, we evaluated the beating of cilia in primary cultured mouse ependymal cells. Live imaging of ependymal cells showed coordinated cilia beatings in WT and decreased cilia motility in *Kif6^pG555fs^* mice (Fig. 3H, Movies 5-7). Consistent with the variable penetrance of hydrocephalus, we found that, at D14, *Kif6^pG555fs^* ependymal cells had normal, faint or no motile cilia (Fig. 3I, Movie 6) and that the beating frequency of ependymal cilia, when present, was significantly decreased in *Kif6^pG555fs^* compared with WT cilia (Fig. 3J). Interestingly, at D21, cilia beat frequency in *Kif6^pG555fs^* mice was not significantly different compared with that in WT mice (Fig. 3J), suggesting that cilia beating in *Kif6^pG555fs^* was restored as ependymal cells mature. Moreover, we found that the percentage of ependymal cells with immotile cilia was decreased in Kif6 mutant mice at D21 (Fig. S4A). The remaining *Kif6^pG555fs^* ependymal cells at D21 had normal apical actin networks (Fig. S4B–D) and better cilium orientation (Fig. S4E,F) than the population of Kif6 mutant ependymal cells at D14. Thus, Kif6 appears to play a stage-dependent role in organizing directional ciliary beating.

As kinesins can form homo- and hetero-dimers, kinesins without the motor domain (motor-less) can act as dominant-negative proteins (Gelfand et al., 2001). To test this model, we transfected a motor-less Kif6 tagged to mCherry at its N-terminal end (hereafter referred to as mCherry-Kif6 ML) into ependymal cells. We observed that ependymal cells at D14 expressing mCherry-Kif6 ML significantly suppressed cilia beatings compared with cells expressing mCherry control (Fig. 3K,L, Movies 8 and 9). These results suggest that Kif6 regulates cilia motility in ependymal cells.

### Ependymal cilia in *Kif6^p.G555fs^* display normal axoneme ultrastructure

**Fig. 4.**
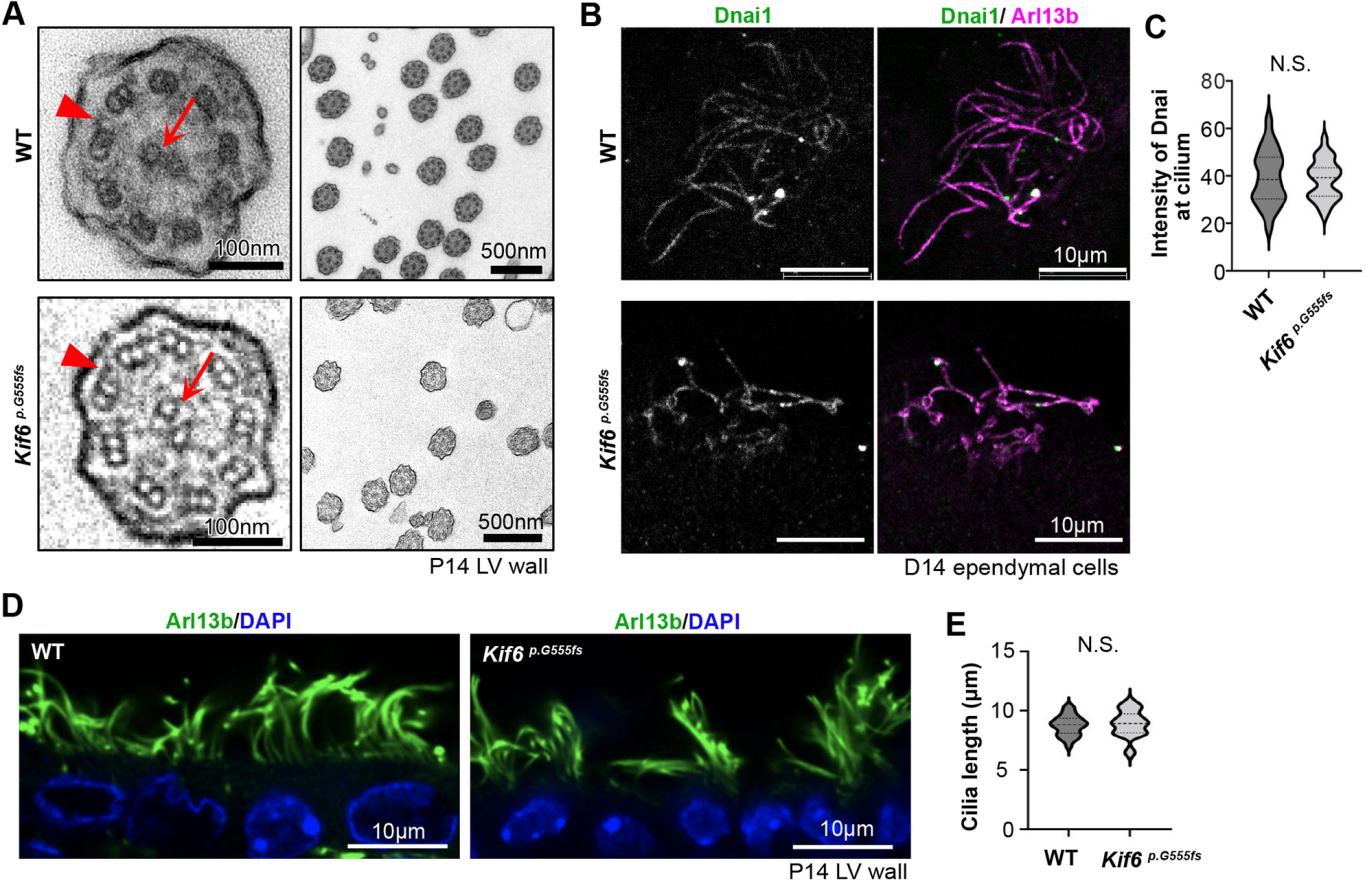
**Ependymal cilia in *Kif6^p.G555fs^* display normal 9+2 architecture.** (A) Representative TEM images of ependymal cilia at P14 WT or *Kif6^p.G555fs^* LV wall. Arrows indicate central pair of microtubules. Arrowheads indicate outer dynein arm. (B) WT or *Kif6^p.G555fs^* ependymal cells at D14 were immunostained with anti-Dnai1 (green, outer dynein arm staining) and anti-Arl13b (magenta, cilia staining). (C) Quantification of Dnai1 intensity at cilia. Mean intensity of Dnai1 was normalized against Arl13b intensity at cilia. The violin plot shows the distribution of Dnai1 staining intensity with median and quartile ranges WT (median=38.53, *n*=48 cilia from three experiments) and *Kif6^p.G555fs^* (median=39.25, *n*=48 cilium from three experiments) mice at P14. *P*-value (0.796) was determined with the Mann–Whitney test. (D) Cross section of ependymal cells in the middle lateral side of LV wall. Vibratome sections from P14 WT or *Kif6^p.G555fs^* brain immunostained for cilia with anti-Arl13b (green), nuclei were stained with DAPI (blue). (E) Graph represents cilia length in WT or *Kif6^p.G555fs^* LV wall. The violin plot shows the distribution of cilia length with median and quartile ranges in WT (median=8.821, *n*=49 cilia from three mice) and P14 *Kif6^p.G555fs^* (median=8.928, *n*=49 cilia from three mice) mice at P14. *P*-value (0.4341) was determined with the Welch's test.

Cilia motility requires a radially arranged structure of nine doublet microtubules and axonemal dyneins in repeating patterns (Ma et al., 2019). To observe if ultrastructural defects may explain the decreased cilia beat frequency, we visualized cilia structures in WT and *Kif6^pG555fs^* mutant mice by using transmission electron microscopy (TEM). TEM images showed a typical 9+2 arrangement – i.e. nine doublet microtubules surrounded by a central pair of singlet microtubules – with outer dynein arms in both WT and *Kif6^pG555fs^* ependymal cilia at postnatal day (P)14 (Fig. 4A, Fig. S5). By using immunofluorescence we show that the outer dynein arm component Dnai1 was present in *Kif6^pG555fs^* ependymal cell cilia as in WT (Fig. 4B,C). Decreased cilia length is associated with decreased cilia motility (Bottier et al., 2019); however, we found that the length of *Kif6^pG555fs^* ependymal cilia, when present, was not significantly different from that of control cilia (Fig. 4D,E). Finding no overt structural defects in *Kif6^pG555fs^* ependymal cilia, we turned our attention to the MCC apical cytoplasm.

### *Kif6* is required for planar polarization of ependymal cells

**Fig. 5.**
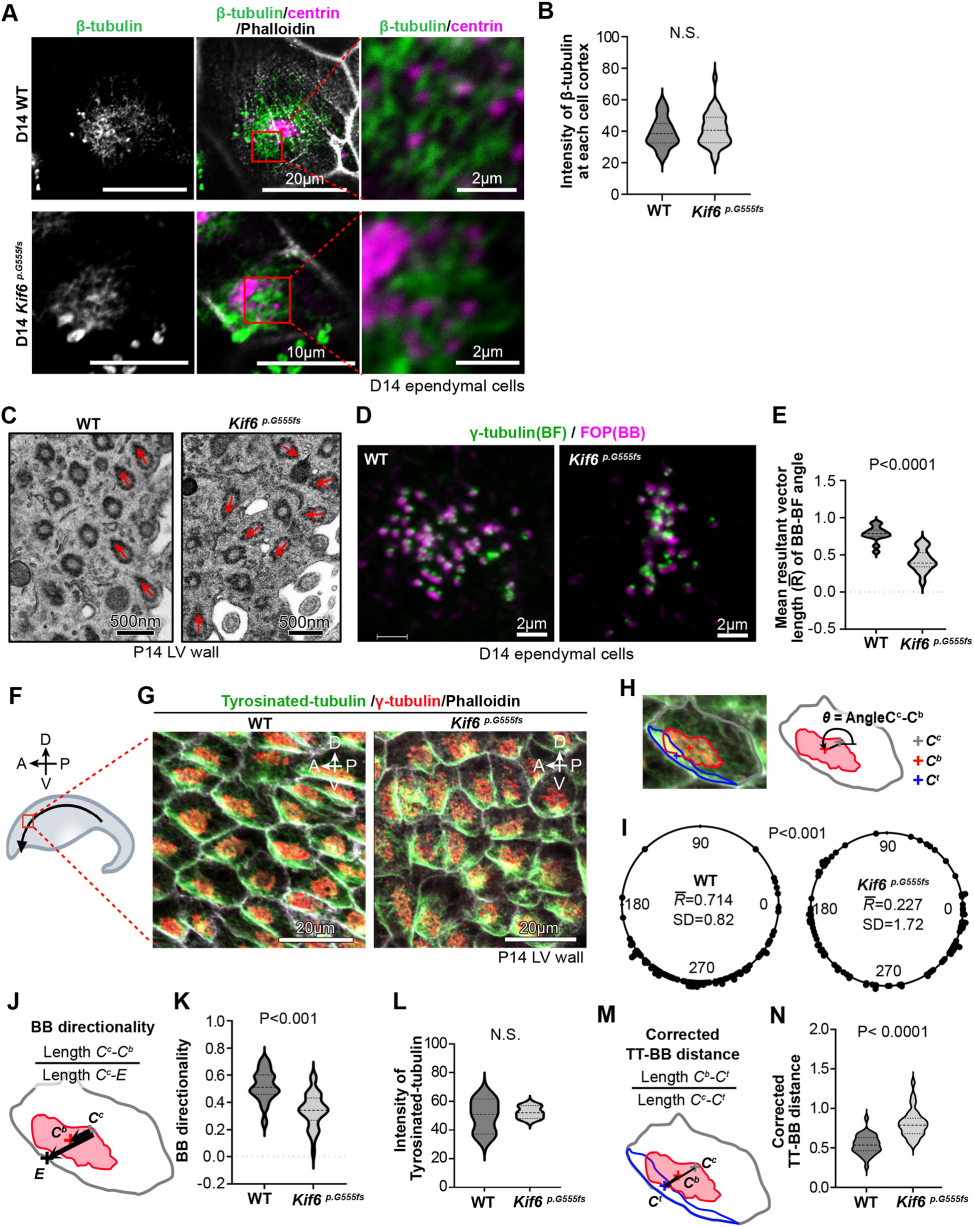
***Kif6^p.G555fs^* is required for planar polarization of ependymal cells.** (A) Representative images of apical microtubules at ciliary base in D14 WT or *Kif6^p.G555fs^* ependymal cells. WT or *Kif6^p.G555fs^* cells are immunostained with anti-β-tubulin (green), anti-centrin (magenta), and phalloidin (gray). Red boxes show microtubules connecting basal bodies (BBs), expanded in the right panels. (B) Violin plot showing the intensity of β-tubulin at apical cell cortex. The violin plot shows the distribution of β-tubulin staining intensity with median and quartile ranges in WT (median=38.52, *n*=37 cells from three LV walls) and *Kif6^p.G555fs^* (median=40.62, *n*=37 cells from three LV walls) mice at P14. *P*-value (=0.36) was determined with the Welch's test. (C) TEM images of ciliary base in ependymal cell from P14 WT or *Kif6^p.G555fs^* LV wall. Arrows indicate vectors from BBs to basal feet (BF). (D) Representative immunostaining images of γ-tubulin (green, as a BF marker) and FOP (magenta, as a BB marker) in D14 WT or *Kif6^p.G555fs^* ependymal cells. (E) Violin plot showing the mean resultant vector length (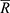) of BB-BF angles that were measured in immunostaining images as shown in D. The violin plot shows the distribution of 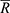 with median and quartile ranges in WT (median=0.79, *n*=15 cells from two experiments) and *Kif6^p.G555fs^* (median=0.39, *n*=15 cells from two experiments) mice at P14. *P*-value was determined with the Welch's test. (F) Schematic of the measurement area to observe translational polarity in mouse LV wall tissue at P14. Whole-mount immunostained LV walls were observed at the anterior area (red box) of LV wall. The bent arrow indicates the direction of the CSF flow. Plane-of-section compass (top left within top left image): A, anterior; P, posterior; D, dorsal; V, ventral. (G) Representative fluorescence microscopy images of whole-mount immunostaining against tyrosinated tubulin (green) and γ-tubulin (red), and for phalloidin (gray) in the anterior area of P14 WT or *Kif6^p.G555fs^* LV walls. (H) Illustration showing the measurement of the BB clustering position in the apical cell cortex of ependymal cells. (I) Vector angles (θ; as shown in H) were calculated from the center of the cell to the center of the multicilia bundle base (BB clustering), i.e. the aggregated region of γ-tubulin dots. Vector angles θ are plotted as circular diagram in P14 WT or *Kif6^p.G555fs^* LV wall tissue. The mean resultant vector length (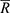) and SD of θ were calculated in WT (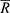
=0.714, *n*=100 cells from three mice) or *Kif6^p.G555fs^* (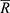
=0.227, *n*=100 cells from three mice). *P*-value was determined with Watson's two-sample test. (J) Illustration showing the measurement of the directionality of BB clustering to the cell border. The distance between the center of the cell and the center of the BB clustering (*C^c^-C^b^*) was divided by the distance between the center of the cell and the cell edge (*C^c^-E*), and defined as ‘BB directionality’. (K) Violin plot showing BB directionality in mouse WT and *Kif6^p.G555fs^* LV walls at P14, measured as shown in J. The violin plot shows the distribution with median and quartile ranges in WT (median=0.51, *n*=56 cells from three mice) and *Kif6^p.G555fs^* (median=0.34, *n*=56 cells from three mice) mice at P14. *P*-value was determined with the Welch's test. (L) Violin plot showing the intensity of tyrosinated tubulin at apical cell cortex as shown in G. The violin plot shows the distribution with median and quartile ranges in WT (median=50.93, *n*=3 mice) and P14 *Kif6^p.G555fs^* (median=52.01, *n*=3 mice) mice at P14. *P*-value (=0.7717) was determined with Welch's test. (M) Illustration showing the measurement of the distance between the polarized tyrosinated tubulin and BB clustering. The distance between the center of BB clustering and the center of the asymmetrically accumulated tyrosinated tubulin area (the polarized tyrosinated tubulin), i.e. *C^b^-C^t^*, was divided by the distance between the center of cell and the center of the polarized tyrosinated tubulin (*C^c^-C^t^*), as defined as ‘Corrected TT-BB distance’. (N) Violin plot showing the corrected TT-BB distance in mouse WT or *Kif6^p.G555fs^* LV walls at P14, measured as shown in M. The violin plot shows the distribution with median and quartile ranges in WT (median=0.537, *n*=34 cells from three mice) and *Kif6^p.G555fs^* (median=0.787, *n*=34 cells from three mice) mice at P14. *P*-value was determined with the Mann–Whitney test.

Because Kif6 was localized to apical cytoplasmic microtubules, we next analyzed the apical microtubules that in ependymal cells form an organized network and align the basal bodies (BB's) (Werner et al., 2011; Kunimoto et al., 2012). Because Kif19A is known to depolymerize microtubules (Niwa et al., 2012), we first examined general microtubule organization in *Kif6^pG555fs^* ependymal cells and found that overexpression of mCherry-Kif6 full length (mCherry-Kif6 FL) or dominant-negative mCherry-Kif6 ML had no effect on acetylated-tubulin, which correlates with stable microtubules in ependymal cell bodies (Fig. S6). Moreover, *Kif6^pG555fs^* did not diminish the intensity of β-tubulin labeled microtubules accumulated around BBs (Fig. 5A,B).

Because the surface flow was disturbed on *Kif6^pG555fs^* LV wall tissue (Fig. 3F), we next analyzed cilia polarity and orientation at single-cell (rotational polarity) and tissue level (translational polarity) (Mirzadeh et al., 2010). Rotational polarity was quantified by the orientation of the BF projection from BB. By using TEM for LV wall tissue at P14, we were able to show that the orientation of the BF-BB is perturbed in *Kif6^pG555fs^* mice compared to the parallel orientation typically observed in WT mice (Fig. 5C). We also observed this rotational polarity defect in cultured ependymal cells derived from *Kif6^pG555fs^* mouse mutants by immunostaining for the BB marker FGFR1 oncogene partner (FOP; officially known as CEP43) and the BF marker γ-tubulin (Fig. 5D). The orientation of BB-BF alignments in the plane of cultured ependymal cells at D14, was calculated as the mean resultant vector length (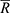) and found to be significantly reduced in *Kif6^pG555fs^* ependymal cells (Fig. 5E).

Ependymal cilia also display translational polarity, visible as the clustering of BB on LV wall tissue along the direction of CSF flow (Ohata and Alvarez-Buylla, 2016). We observed BB clustering within each ependymal cell at the anterior–dorsal area of LV wall tissue, finding them positioned ventrally (Fig. 5F). This translational polarity was disturbed in *Kif6^pG555fs^* cells (Fig. 5G). BB clustering was quantified as vector angles from the center of the cell to the center of the cilium bundle base (Fig. 5H), and found to be more randomly distributed in *Kif6^pG555fs^* LV wall tissue compared with the polarized distribution observed in WT tissue (Fig. 5I). BB clusters in *Kif6^pG555fs^* mutants were close to the center of cell (Fig. 5J) and the directionality was significantly decreased (Fig. 5K).

It has been previously shown that tyrosinated tubulin, which indicates newly polymerized microtubule plus ends, is asymmetrically localized to apical junctions in ependymal cells, and that this microtubule polarization promotes cilia polarity (Takagishi et al., 2020). However, we found that *Kif6^pG555fs^* LV wall tissue showed the normal asymmetric localization of tyrosinated tubulin (Fig. 5G). We also observed no difference in the intensity of staining for tyrosinated tubulin between *Kif6^pG555fs^* and WT LV wall tissue (Fig. 5L). By contrast, the directionality of BB clusters towards polarized tyrosinated tubulin was significantly decreased in *Kif6^pG555fs^* LV wall tissue (Fig. 5M,N). These results suggest that Kif6 affects cilia polarity independently of the apical microtubules.

### Loss of Kif6 function affects apical actin assembly and BB stability

**Fig. 6.**
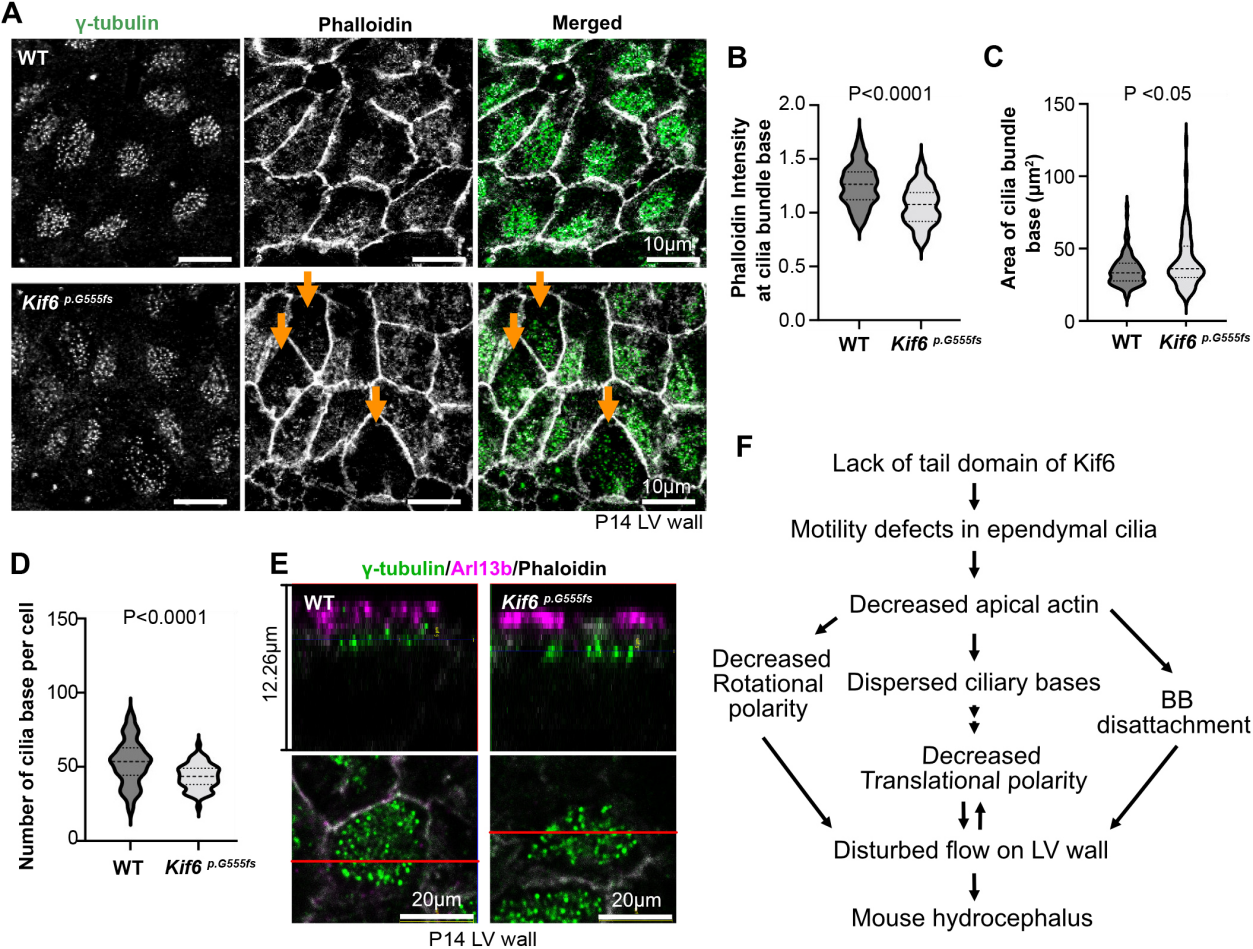
***Kif6^p.G555fs^* decreases apical actin and basal body stability.** (A) Representative images of whole-mount immunofluorescence staining for γ-tubulin (green) and phalloidin (gray) to mark actin in the anterior area of WT or *Kif6^p.G555fs^* LV walls at P14. Arrows indicate cells with decreased phalloidin staining and dispersed ciliary bases, i.e. basal bodies (BBs). (B) Plotted is the phalloidin intensity at the cilia bundle base. The intensity of phalloidin at γ-tubulin dots area was divided by the intensity of phalloidin at the apical cell cortex. The violin plot shows the distribution of phalloidin intensity with median and quartile ranges in WT (median=1.26, *n*=100 cells from three mice) and P14 *Kif6^p.G555fs^* (median=1.08, *n*=99 cells from three mice) mice at P14. *P*-value was determined with the Welch's test. (C) Plotted is the spreading area of γ-tubulin dots as the area of cilia bundle bases. The violin plot shows the area of the cilia bundle base with median and quartile ranges in WT (median=33.21, *n*=100 cells from three mice) and *Kif6^p.G555fs^* (median=36.08, *n*=101 cells from three mice) mice at P14. *P*-value (0.0162) was determined with the Mann–Whitney test. (D) Plotted is the number of γ-tubulin dots in the apical cell cortex as the number of ciliary bases per cell. The violin plot shows their distribution with median and quartile ranges in WT (median=53.50, *n*=60 cells from three mice) and *Kif6^p.G555fs^* (median=43.5, *n*=60 cells from three mice) mice at P14. *P*-value was determined with the Mann–Whitney test. (E) Representative confocal microscopy images of whole-mount immunofluorescence staining with γ-tubulin (green), Arl13b (magenta) and phalloidin (gray) in WT or *Kif6^p.G555fs^* mouse LV walls at P14. Top panels show the cross section of the ependymal cells that are depicted in the bottom panels. Positions of the cross section are indicated by red lines. (F) Effects of cilia disfunction in *Kif6^p.G555fs^* ependymal cells on mouse LV wall tissue. Lack of the Kif6 tail domain (i.e. mutant *Kif6^p.G555fs^*) causes motility defects in ependymal cells during brain development. Motility defects in ependymal cilia reduce the formation of the apical actin network. Decreased apical actin results in the destabilization of the ciliary base, leading to decreased rotational polarity, dispersed ciliary bases and detachment of BBs, which – in turn – contributes to a disturbed flow on the LV wall and to mouse hydrocephalus.

Apical actin forms networks in MCCs, which aid in connecting BBs and contribute to their alignment, orientation and stabilization in MCCs (Werner et al., 2011; Mahuzier et al., 2018). By using phalloidin conjugated to a fluorescent dye to label actin filaments, we observed colocalization of accumulated apical actin with BBs in both WT and *Kif6^pG555fs^* LV wall tissues (Fig. 6A). However, *Kif6^pG555fs^* wall tissue showed a significant decrease in actin accumulation at with BB clusters – measured as phalloidin signal intensity at the cilia bundle base (Fig. 6B). Moreover, the area of BB clusters was significantly increased in *Kif6^pG555fs^* ependymal cells (Fig. 6C) and dispersed across the apical cell cortex with decreased actin, compared to the tight accumulation of BBs in WT ependymal cells (Fig. 6A). Importantly, the loss of apical actin networks has been shown to induce BB dis-attachment in ependymal cells (Mahuzier et al., 2018). Accordingly, we found that the number of cilium bases, as counted by the number of BBs significantly decreased in *Kif6^pG555fs^* ependymal cells (Fig. 6D). Because the BBs were not found in deeper cytoplasm (Fig. 6E), these results suggest that the defect of apical actin networks in *Kif6^pG555fs^* cause destabilization of BBs and shedding of ependymal cilia.

## DISCUSSION

Here, we described a complex phenotype of cilia-related defects in the ependymal cells of Kif6 mutant mice. We suggested that this complex phenotype emerges from pleiotropic defects in ependymal cells. Because we found that Kif6 actively moves along microtubules and is present in axonemes (Fig. 1), defects in cilia beating may result from the direct action of Kif6 in cilia motility. By contrast, because we demonstrated that Kif6 is required for apical actin assembly, its presence in the apical cytoplasm may explain defects in cilia polarity. Finally, we suggested that the defects in apical actin assembly also result in the loss of cilia from ependymal cells. Together, this constellation of ciliary defects results in defective CSF and moderate hydrocephalus in Kif6 mutant mice (Fig. 6F).

We have shown here that the kinesin-9B family member Kif6 is a slow processive motor *in vitro*, yet were unable to observe processive movement of the protein within cilia *in vivo*. Therefore, Kif6 closely resembles the related kinesin-9A protein Kif9 , as it undergoes slow processive motility *in vitro* but not inside cilia (Konjikusic et al., 2023). In addition, processive movement in axonemes of another kinesin-9A family member, i.e. the *Chlamydomonas* protein Klp1, also have not been observed (Han et al., 2022). One possibility is that the slow motility of kinesin-9 proteins evolved to permit nucleotide-dependent engagement and disengagement with central-pair microtubules and, thereby, contribute to ciliary bending in a manner analogous to how the non-processive axonemal dyneins engage with doublet microtubules (Han et al., 2022). Alternatively, the motility of kinesin-9 motor proteins in axonemes may be hindered, given that the central-pair microtubules are decorated by a tight array of associated proteins that do not appear to leave any unoccupied surface for processive movement of a kinesin (Yokoyama et al., 2004).

The defects in cilia motility reported here in Kif6 mutant mice may relate to the cilia motility defects observed after knockdown of *Trypanosoma KIF9B,* which disrupts beating and formation of the paraflagellar rod (PFR) within the flagellar membrane of *T*. *brucei* (Demonchy et al., 2009). However, since mammalian ependymal cilia do not have an analogous PFR structure, the precise location of Kif6 action within the vertebrate axoneme remains to be determined. One possibility is that Kif6 acts in the central pair, similar to the kinesin-9A proteins Kif9 in vertebrates and KLP1 in *C. reinhardtii* (Konjikusic et al., 2023; Han et al., 2022). This possibility is supported by recent mapping of the protein interactome in *Tetrahymena thermophila* by using cross-linking mass spectrometry, which identified links between Kif6 and the central-pair protein Spag16 and the nearby radial spoke-head protein Rsph4 (McCafferty et al., 2023 preprint). In fact, some redundancy between Kif6 and Kif9 might explain the finding that *Kif6^p.G555fs^* ependymal cilia display decreased motility at D14 but recovered by D21 (Fig. 1J).

Kif6 mutation did not only affect cilia motility but also cilia polarity (Fig. 5), and these polarity defects are likely to contribute to the disturbed flow over LV wall tissue (Fig. 3F). Cilium orientation is regulated by apical microtubules (Vladar et al., 2012; Takagishi et al., 2020) and affected by apical actin networks between BBs (Werner et al., 2011), so Kif6 localization to this region is of interest as it is clearly distinct from that observed for Kif9 (Konjikusic et al., 2023). However, Kif6 mutation did not affect the overall appearance of apical microtubules (Fig. 5, Fig. S6), suggesting disturbed cilia polarity is secondary to the defects in cilia motility.

Indeed, cilia beating motility induces apical actin network organization that is crucial for BB stabilization (Mahuzier et al., 2018). Suppressed cilia motility would be expected to result in decreased apical actin networks around BBs, and destabilized BBs could be detached (Mahuzier et al., 2018). Because we observed all of these phenotypes it is possible that, despite the presence of Kif6 in the apical cytoplasm, Kif6-related actin defects are secondary to cilia-beating defects. A final possibility is that, because of BB destabilization, Kif6 mutant ependymal cilia with decreased motility are physically removed (by shedding), as suggested by Mahuzier et al., 2018 (Fig. 6D–F), and that stable cilia that remain until D21 display normal beating.

## MATERIALS AND METHODS

### Mutant mice

*Kif6^pG555fs^* mutant mice were generated using the CRISPR-Cas9 system (Konjikusic et al., 2018). *Kif6^pG555fs^* or C57Bl/6J mice were used for whole-mount staining, ependymal cell culture and LV wall tissue explant cultures. All mice were maintained at a 12/12 h light/dark cycle with no more than five mice per cage. Both male and female mice were used for the experiments, and sex differences were not observed. All mouse experiments were approved by the Animal Studies Committee at University of Texas at Austin (AUP-2021-00118).

### Plasmids

Mouse Kif6 (mKif6) cDNA encoding the 803 aa protein (NP_796026) was cloned from a mouse brain cDNA library and sequenced. The cDNAs of full-length (FL) mKif6 (1-803aa) and motor-less (ML) mKif6 (360-803aa) were then subcloned into pEGFP-C1 vector (Addgene plasmid #54759) or pmCherry2-C1 vector (Addgene plasmid #54563; both a gift from Michael Davidson), yielding EGFP-Kif6 FL or mCherry-Kif6 FL or mCherry-Kif6 ML, respectively. mCherry was removed and mKif6 (1-493aa)-mNeonGreen was added into vector pmCherry2-C1 yielding mKif6 (1-493)-mNG.

### Cell culture, transfection and lysate preparation

The COS-7 (African green monkey kidney fibroblast-like) cell line was obtained from the American Type Culture Collection (ATCC; RRID: CVCL_0224), with cells cultured in DMEM (Gibco) with 10% (vol/vol) FetalClone III serum (HyClone) and 1% GlutaMAX supplement (Gibco) at 37°C under 5% CO_2_. COS-7 cells were transfected with Trans-IT LT1 (Mirus Bio) according to the manufacturer's instructions.

COS-7 cells were collected 48 h post transfection. The cells were harvested by low-speed centrifugation at 1500 ***g*** for 5 min at 4°C. The pellet was rinsed once in PBS and resuspended in ice-cold lysis buffer (25 mM HEPES/KOH, 115 mM potassium acetate, 5 mM sodium acetate, 5 mM MgCl_2_, 0.5 mM EGTA, and 1% Triton X-100 pH 7.4) freshly supplemented with 1 mM ATP, 1 mM phenylmethylsulfonyl fluoride, and protease inhibitor cocktail (#P8340; Sigma-Aldrich). After the lysate was clarified by centrifugation at 20,000 ***g*** for 10 min at 4°C, aliquots of the supernatant were snap-frozen in liquid nitrogen and stored at −80°C until further use.

### Single-molecule motility assays

HiLyte647-labeled microtubules were polymerized from purified tubulin including 10% Hily647-labeled tubulin (cytoskeleton) in BRB80 buffer (80 mM PIPES/KOH pH 6.8, 1 mM MgCl_2_, and 1 mM EGTA) supplemented with 1 mM GTP and 2.5 mM MgCl_2_ at 37°C for 30 min. 20 μM taxol in pre-warmed BRB80 buffer was added and incubated at 37°C for additional 30 min to stabilize microtubules. Microtubules were stored in the dark at room temperature (RT) for further use.

A flow cell (∼10 μl volume) was assembled by attaching a clean 1.5-mm thick coverslip (#1.5 Fisher Scientific) to a glass slide (Fisher Scientific) with two strips of double-sided tape. Polymerized microtubules were diluted in BRB80 buffer supplemented with 10 μM taxol, infused into flow cells and incubated for 5 min at RT for nonspecific adsorption to the coverslips. Subsequently, blocking buffer [15 mg/ml BSA in P12 buffer (12 mM PIPES/KOH pH 6.8, 1 mM MgCl_2_, and 1 mM EGTA)] was infused and incubated for 5 min. Finally, KIF6 cell lysate in the motility mixture [2 mM ATP, 0.4 mg/ml casein, 6 mg/ml BSA, 10 μM taxol, and oxygen scavenger (1 mM DTT, 1 mM MgCl_2_, 10 mM glucose, 0.2 mg/ml glucose oxidase, and 0.08 mg/ml catalase) in P12 buffer] was added to the flow cells. The flow cell was sealed with molten paraffin wax. To examine the movement direction of zebrafish and mouse Kif6 (dKif6 and mKif6, respectively), motility buffer containing the cell lysates of Kif6-mNG and KIF1A(1-393)-Halo^JFX554^ was added into the flow cell, and the flow cell was sealed and imaged.

Images were acquired by total internal reflection fluorescence (TIRF) microscopy using an inverted microscope Ti-E/B equipped with the perfect focus system (Nikon), a 100×1.49 NA oil immersion TIRF objective (Nikon), three 20-mW diode lasers (488 nm, 561 nm and 640 nm) and an electron-multiplying charge-coupled device detector (iXon X3DU897; Andor Technology). Image acquisition was controlled using Nikon Elements software and all assays were performed at RT. Images were acquired, at a rate of one frame every 3 s for 3 min for mKif6, or at a rate of one frame every 100 ms for 10 s for KIF1A.

Maximum-intensity projections were generated, and the kymographs were produced by drawing along tracks of motors (width= three pixels) using Fiji/ImageJ2 . Motors frequently paused during motility events and, thus, velocity between pauses was analyzed. Velocity was defined as the distance of the kymograph (on the *x*-axis) divided by the time of the kymograph (on the *y*-axis).

### Ependymal cell culture

LV wall tissues from three or four mouse brains at postnatal days 0 or 1 (P0 or P1, respectively) were collected and the suspended cells with trypsin-EDTA were cultured in DMEM with 10% FBS for 3 days in a laminin-coated flask. After shaking for 2 h, the adherent cells were plated on coverslips coated with poly-L-lysine and laminin, and differentiated into ciliated ependymal cells (ependymal cells) by serum starvation. Ependymal cells were then transfected with mKif6(1-493)-mNG or mCherry-Kif6 constructs using Lipofectamine 2000 (Invitrogen) 48 h before experiments.

### Live cell imaging

For live imaging of Kif6(1-493)-mNG, D5 ependymal cells were transfected with Kif6(1-493)-mNG at D5 and imaged at D7 for 5 min in 0.2 frames per second (FPS), using a Ti2E inverted microscope with high-content screening system (Nikon). To observe cilia beatings, ependymal cells were plated on glass bottom dishes (20 mm, Cellvis). Time-lapse and digital image correlation (DIC) images were collected for 2 s at 47, 95 or 190 FPS by using a Ti2E microscope with a high-content screening system (Nikon). Ciliary tips were tracked using the manual tracking plug-in within Fiji for images shown in Fig. 3H and K. Kymographs shown in Fig. 3I were generated along the track of ciliary tips at 47 FPS for 200 ms. The duration of one beat (round-trip) of ciliary tips was measured in kymographs. Ciliary beating frequency (in Hz) was calculated as beats per second. Immotile cilia, as shown in Fig. 3I (bottom panel), were counted as 0.

### Western blotting

LV wall tissues were collected from three mice each at P0, P4, P7 or P14. Each LV wall tissue or P45 testis tissue was lysed in PHEM buffer (50 mM PIPES, 50 mM HEPES, 1 mM EDTA, 2 mM MgSO4, 1% Triton X-100) with protease inhibitors, homogenized, and centrifuged for 1 h at 17800 ***g***. The lysate supernatant was mixed with Laemmli sample buffer (Bio-Rad) and 2-mercaptoethanol and boiled. Samples were separated by SDS-PAGE and transferred to nitrocellulose membranes. The membranes were blocked with 2% BSA and probed with anti-Kif6 (1:1000, Proteintech, 17290-I-AP) or anti-GAPDH (1:1000, Cell Signaling Technology, #2118) antibodies. Detection was carried out using HRP-linked anti-Rabbit IgG (1:2000, Cell signaling Technology, #7074) and visualized using Pierce ECL substrate (Thermo Fisher Scientific).

### H&E staining and immunostaining

Mouse brains were fixed in 4% paraformaldehyde at 4°C for overnight. The fixed whole brains were paraffin-embedded and cut into 5 µm sections for hematoxylin & eosin (H&E) staining. To measure cilia length, the fixed brains were sectioned in the coronal plane at 100 µm on a vibratome and immunostained. Whole-mount preparations of LV wall tissue were fixed with cold methanol for 10 min and 4% paraformaldehyde for overnight at 4°C. Cultured ependymal cells were fixed with cold methanol for 3 min and 4% paraformaldehyde for 10 min at RT. Whole-mount, ependymal cell or vibratome-sectioned samples were blocked with 10% goat serum in PBS for 30 min and incubated with primary antibodies for overnight at 4°C. Primary antibodies, were anti-Kif6 (1:100, Proteintech, 17290-I-AP), anti-Arl13b (1:200, Proteintech, 17711-I-AP), anti-Acetylated Tubulin (1:200, Sigma, T7451), anti-gamma Tubulin (1:100, Abcam, ab11321), anti-beta Tubulin (1:100, Abcam, ab7287), anti-Centrin (1:50, Sigma, 04-1624), anti-FGFR1OP (1:100, Proteintech, 11343-1-AP), anti-Dnai1 (1:100, NeuroMab, 73-372), or anti-Tubulin [YL 1/2] (1:200, Abcam, ab6160), were detected with secondary antibodies conjugated to Alexa Fluorophores (Invitrogen). The apical surface of ependymal cells was imaged using a confocal laser scanning microscope LSM700 (Zeiss) or LSM 980 Airyscan 2 SR mode with joint Deconvolution (Nikon) for super-resolution images. H&E or immunostained tissue sections were imaged using a BZ-X710 fluorescence microscope (KEYENCE).

### Bead migration on LV wall tissue

The lateral sides of LV wall tissue were dissected from mouse brain at P12 and pinned on a silicon plate covered with DMEM. Fluorescent microsphere (1 µm) -containing PBS was placed on the middle part of LV walls. Bead migration was recorded in ten FPS for 10 s using an AXIO Zoom.V16 microscope (Zeiss). Time-projection images were created from 100 frames obtained by using Fiji software (Schindelin et al., 2012). Beads migration speed was calculated by using the manual tracking plug-in of Fiji.

### Electron microscopy

Mouse LV wall tissues obtained at P14 were fixed overnight using 3% glutaraldehyde with 2% paraformaldehyde. Tissues were post fixed in 1% osmium tetroxide with 1% potassium ferrocyanide for 3 h. After en-bloc staining with 1% aqueous uranyl acetate for 1 h, ethanol-dehydrated tissues were displaced into aceton and embedded in epoxy resin. Tissues were sectioned (80 nm thickness) with an UltraCut Ultramicrotome (Leica). Images of the apical cell cortex of ependymal cells were obtained using a Tecnai Transmission Electron Microscope (FEI).

### Statistical analyses

The violin plots were generated using GraphPad Prism 9 (GraphPad) Welch's *t*-test or Mann–Whitney *U*-test were used to compare the means of two experimental groups by using GraphPad Prism. Differences were considered statistically significant at *P*<0.05. To determine the rotational BB orientation for a single ependymal cell (Fig. 5E), a vector was drawn from the center of a FOP dot to the center of the closest γ-tubulin dot in each cell; more than ten vectors were averaged. Vector angles were measured using Fiji and plotted as a circular diagram using the statistical software R (R Core Team, 2019: https://www.r-project.org/). Mean resultant vector lengths (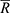) were calculated using the ‘circular’ package (https://cran.r-project.org/web/packages/circular/index.html) within the R statistical computing environment. For Fig. 5I, vector angle distribution of cilia bundles on WT and *Kif6^pG555fs^* LV wall tissue was compared by using Watson's two-sample U2 test (R software).

## Supplementary Material

10.1242/dmm.050137_sup1 Supplementary information

## References

[DMM050137C1] Assimes, T. L., Hólm, H., Kathiresan, S., Reilly, M. P., Thorleifsson, G., Voight, B. F., Erdmann, J., Willenborg, C., Vaidya, D., Xie, C. et al. (2010). Lack of association between the Trp719Arg polymorphism in kinesin-like protein-6 and coronary artery disease in 19 case-control studies. *AJ. Am. Coll. Cardiol.* 56, 1552-1563. 10.1016/j.jacc.2010.06.022 PMC3084526 20933357

[DMM050137C3] Bottier, M., Thomas, K. A., Dutcher, S. K. and Bayly, P. V. (2019). How does cilium length affect beating? *Biophys. J.* 116, 1292-1304. 10.1016/j.bpj.2019.02.012 30878201 PMC6451027

[DMM050137C4] Brooks, E. R. and Wallingford, J. B. (2014). Multiciliated cells. *Curr. Biol.* 24, R973-R982. 10.1016/j.cub.2014.08.047 25291643 PMC4441396

[DMM050137C5] Demonchy, R., Blisnick, T., Deprez, C., Toutirais, G., Loussert, C., Marande, W., Grellier, P., Bastin, P. and Kohl, L. (2009). Kinesin 9 family members perform separate functions in the trypanosome flagellum. *J. Cell Biol.* 187, 615-622. 10.1083/jcb.200903139 19948486 PMC2806587

[DMM050137C6] Gelfand, V. I., Bot, N. L., Tuma, M. C. and Vernos, I. (2001). A dominant negative approach for functional studies of the kinesin ii complex. *Methods Mol. Biol.* 164, 191-204. 11217607 10.1385/1-59259-069-1:191

[DMM050137C7] Gennerich, A. and Vale, R. D. (2009). Walking the walk: how kinesin and dynein coordinate their steps. *Curr. Opin. Cell Biol.* 21, 59-67. 10.1016/j.ceb.2008.12.002 19179063 PMC2668149

[DMM050137C8] Gray, R. S., Gonzalez, R., Ackerman, S. D., Minowa, R., Griest, J. F., Bayrak, M. N., Troutwine, B., Canter, S., Monk, K. R., Sepich, D. S. et al. (2021). Postembryonic screen for mutations affecting spine development in zebrafish. *Dev. Biol.* 471, 18-33. 10.1016/j.ydbio.2020.11.009 33290818 PMC10785604

[DMM050137C9] Hammond, J. W., Cai, D., Blasius, T. L., Li, Z., Jiang, Y., Jih, G. T., Meyhofer, E. and Verhey, K. J. (2009). Mammalian kinesin-3 motors are dimeric In Vivo and move by processive motility upon release of autoinhibition. *PLoS Biol.* 7, e72. 10.1371/journal.pbio.1000072 19338388 PMC2661964

[DMM050137C10] Han, L., Rao, Q., Yang, R., Wang, Y., Chai, P., Xiong, Y. and Zhang, K. (2022). Cryo-EM structure of an active central apparatus. *Nat. Struct. Mol. Biol.* 5, 472-482. 10.1038/s41594-022-00769-9 PMC9113940 35578022

[DMM050137C11] Ishikawa, T. (2017). Axoneme structure from motile cilia. *Cold Spring Harb. Perspect Biol.* 9, a028076. 27601632 10.1101/cshperspect.a028076 PMC5204319

[DMM050137C12] Konjikusic, M. J., Yeetong, P., Boswell, C. W., Lee, C., Roberson, E. C., Ittiwut, R., Suphapeetiporn, K., Ciruna, B., Gurnett, C. A., Wallingford, J. B. et al. (2018). Mutations in Kinesin family member 6 reveal specific role in ependymal cell ciliogenesis and human neurological development. *PLoS Genet.* 14, e1007817. 10.1371/journal.pgen.1007817 30475797 PMC6307780

[DMM050137C13] Konjikusic, M. J., Gray, R. S. and Wallingford, J. B. (2021). The developmental biology of kinesins. *Dev. Biol.* 469, 26-36. 10.1016/j.ydbio.2020.09.009 32961118 PMC10916746

[DMM050137C14] Konjikusic, M. J., Lee, C., Yue, Y., Shrestha, B. D., Nguimtsop, A. M., Horani, A., Brody, S., Prakash, V. N., Gray, R. S., Verhey, K. J. et al. (2023). Kif9 is an active kinesin motor required for ciliary beating and proximodistal patterning of motile axonemes. *J. Cell Sci.* 136, jcs259535. 10.1242/jcs.259535 35531639 PMC9357393

[DMM050137C15] Kunimoto, K., Yamazaki, Y., Nishida, T., Shinohara, K., Ishikawa, H., Hasegawa, T., Okanoue, T., Hamada, H., Noda, T., Tamura, A. et al. (2012). Coordinated ciliary beating requires Odf2-mediated polarization of basal bodies via basal feet. *Cell* 148, 189-200. 10.1016/j.cell.2011.10.052 22265411

[DMM050137C18] Mahuzier, A., Shihavuddin, A., Fournier, C., Lansade, P., Faucourt, M., Menezes, N., Meunier, A., Garfa-Traoré, M., Carlier, M. F., Voituriez, R. et al. (2018). Ependymal cilia beating induces an actin network to protect centrioles against shear stress. *Nat. Commun.* 9, 2279. 10.1038/s41467-018-04676-w 29891944 PMC5996024

[DMM050137C19] Ma, M., Stoyanova, M., Rademacher, G., Dutcher, S. K., Brown, A. and Zhang, R. (2019). Structure of the decorated ciliary doublet microtubule. *Cell* 179, 909-922.e12. 10.1016/j.cell.2019.09.030 31668805 PMC6936269

[DMM050137C20] McCafferty, C. L., Papoulas, O., Lee, C., Bui, K. H., Taylor, D. W., Marcotte, E. M. and Wallingford, J. B. (2023). An amino acid-resolution interactome for motile cilia illuminates the structure and function of ciliopathy protein complexes. *bioRxiv*. 10.1101/2023.07.09.548259 PMC11945580 39674175

[DMM050137C21] Mirzadeh, Z., Han, Y. G., Soriano-Navarro, M., García-Verdugo, J. M. and Alvarez-Buylla, A. (2010). Cilia organize ependymal planar polarity. *J. Neurosci.* 30, 2600-2610. 10.1523/JNEUROSCI.3744-09.2010 20164345 PMC2873868

[DMM050137C38] Niwa, S., Nakajima, K., Miki, H., Minato, Y., Wang, D. and Hirokawa, N. (2012). KIF19A is a microtubule-depolymerizing kinesin for ciliary length control. *Dev. Cell.* 22, 1167-1175. 10.1016/j.devcel.2012.10.016 23168168

[DMM050137C23] Ohata, S. and Alvarez-Buylla, A. (2016). Planar organization of multiciliated ependymal (E1) cells in the brain ventricular epithelium. *Trends Neurosci.* 39, 543-551. 10.1016/j.tins.2016.05.004 27311928 PMC5312752

[DMM050137C24] Piddini, E., Schmid, J. A., de Martin, R. and Dotti, C. G. (2001). The Ras-like GTPase Gem is involved in cell shape remodelling and interacts with the novel kinesin-like protein KIF9. *EMBO J.* 20, 4076-4087. 10.1093/emboj/20.15.4076 11483511 PMC149163

[DMM050137C25] Prevo, B., Scholey, J. M. and Peterman, E. J. G. (2017). Intraflagellar transport: mechanisms of motor action, cooperation, and cargo delivery. *FEBS J.* 284, 2905-2931. 10.1111/febs.14068 28342295 PMC5603355

[DMM050137C26] Roberson, E. C., Tran, N. K., Konjikusic, M. J., Fitch, R. D., Gray, R. S. and Wallingford, J. B. (2020). A comparative study of the turnover of multiciliated cells in the mouse trachea, oviduct, and brain. *Dev. Dyn.* 249, 898-905. 10.1002/dvdy.165 32133718 PMC7641511

[DMM050137C27] Ruiz-Ramos, D., Hernández-Díaz, Y., Tovilla-Zárate, C. A., Juárez-Rojop, I., López-Narváez, M. L., González-Castro, T. B., Torres-Hernández, M. E. and Baños-González, M. A. (2015). The Trp719Arg polymorphism of the KIF6 gene and coronary heart disease risk: systematic review and meta-analysis. *Hereditas* 152, 3. 10.1186/s41065-015-0004-7 28096762 PMC5224589

[DMM050137C39] Schindelin, J., Arganda-Carreras, I., Frise, E., Kaynig, V., Longair, M., Pietzsch, T., Preibisch, S., Rueden, C., Saalfeld, S., Schmid, B. et al. (2012). Fiji: an open-source platform for biological-image analysis. *Nat. Methods.* 9, 676-682. 10.1038/nmeth.2019 22743772 PMC3855844

[DMM050137C30] Spassky, N. and Meunier, A. (2017). The development and functions of multiciliated epithelia. *Nat. Rev. Mol. Cell Biol.* 18, 423-436. 10.1038/nrm.2017.21 28400610

[DMM050137C31] Soppina, V., Norris, S. R., Dizaji, A. S., Kortus, M., Veatch, S., Peckham, M. and Verhey, K. J. (2014). Dimerization of mammalian kinesin-3 motors results in superprocessive motion. *Proc. Natl. Acad. Sci. USA* 111, 5562-5567. 10.1073/pnas.1400759111 24706892 PMC3992690

[DMM050137C32] Takagishi, M., Esaki, N., Takahashi, K. and Takahashi, M. (2020). Cytoplasmic dynein functions in planar polarization of basal bodies within ciliated cells. *iScience* 23, 101213. 10.1016/j.isci.2020.101213 32535020 PMC7300155

[DMM050137C33] Verhey, K. J. and Hammond, J. W. (2009). Traffic control: regulation of kinesin motors. *Nat. Rev. Mol. Cell Biol.* 10, 765-777. 10.1038/nrm2782 19851335

[DMM050137C34] Vladar, E. K., Bayly, R. D., Sangoram, A. M., Scott, M. P. and Axelrod, J. D. (2012). Microtubules enable the planar cell polarity of airway cilia. *Curr. Biol.* 22, 2203-2212. 10.1016/j.cub.2012.09.046 23122850 PMC3518597

[DMM050137C35] Werner, M. E., Hwang, P., Huisman, F., Taborek, P., Yu, C. C. and Mitchell, B. J. (2011). Actin and microtubules drive differential aspects of planar cell polarity in multiciliated cells. *J. Cell Biol.* 195, 19-26. 10.1083/jcb.201106110 21949415 PMC3187709

[DMM050137C36] Wickstead, B., Gull, K. and Richards, T. A. (2010). Patterns of kinesin evolution reveal a complex ancestral eukaryote with a multifunctional cytoskeleton. *BMC Evol. Biol.* 10, 110. 10.1186/1471-2148-10-110 20423470 PMC2867816

[DMM050137C37] Yokoyama, R., O'toole, E., Ghosh, S. and Mitchell, D. R. (2004). Regulation of flagellar dynein activity by a central pair kinesin. *Proc. Natl. Acad. Sci. U.S.A.* 101, 17398-17403. 10.1073/pnas.0406817101 15572440 PMC536025

