# Supplementary material for: Motor protein Kif6 regulates cilia motility and polarity in brain ependymal cells
Source: Dis Model Mech. 2024 Feb 29;17(2):dmm050137. doi: 10.1242/dmm.050137 (PMC10924229; doi:10.1242/dmm.050137)
Supplement: Supplementary information [file dmm-17-050137-s1.pdf]

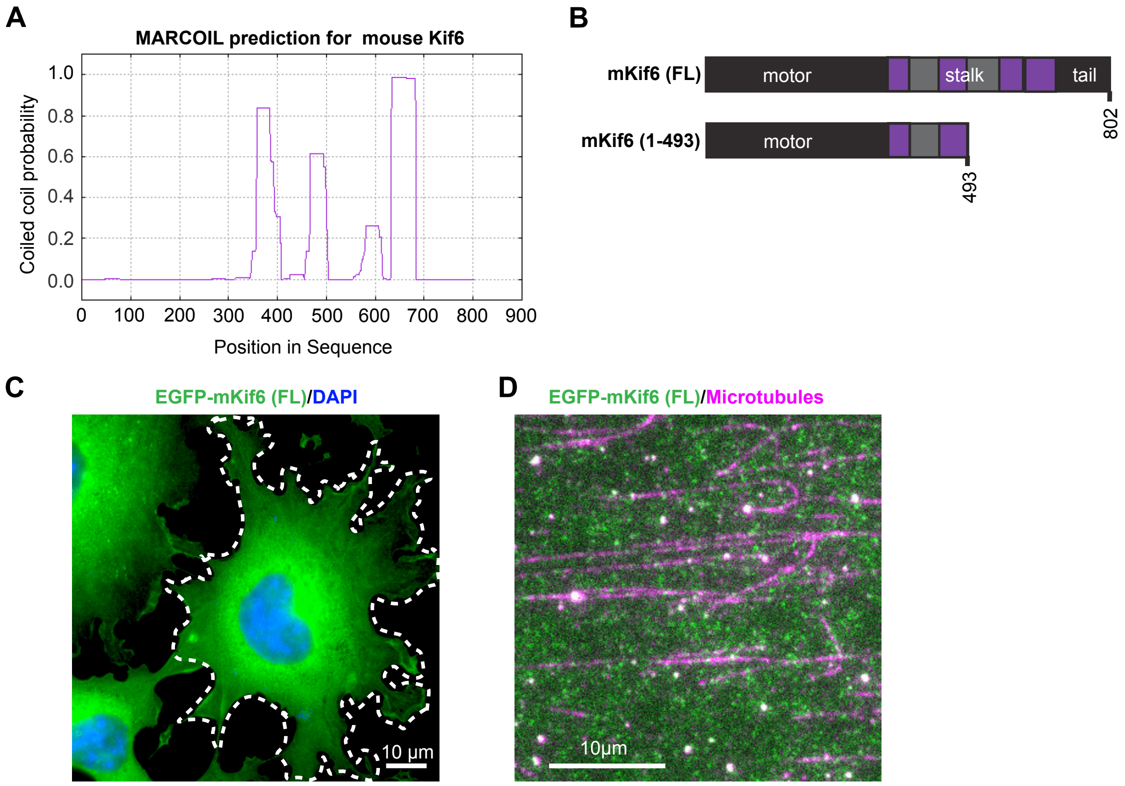

Fig. S1. Full-length of Kif6 is regulated by autoinhibition, related to Fig. 1.

(A) Coiled-coil predictions of mouse Kif6 using Marcoil prediction software (Delorenzi and Speed. 2002. Bioinformatics).

(B) Schematic of the domain composition of full-length and truncated active proteins.

(C) Representative images of EGF-mKif6 FL expressed in COS-7 cells. White dashed lines indicate the outline of a transfected cell. Blue, DAPI stain. Scale bar, 10 µm.

(D) Representative still images from single-molecule motility assays of mKif6(1-493)-mNG on taxol-stabilized microtubules (magenta). Scale bar, 10 µm.

Disease Models & Mechanisms • Supplementary information

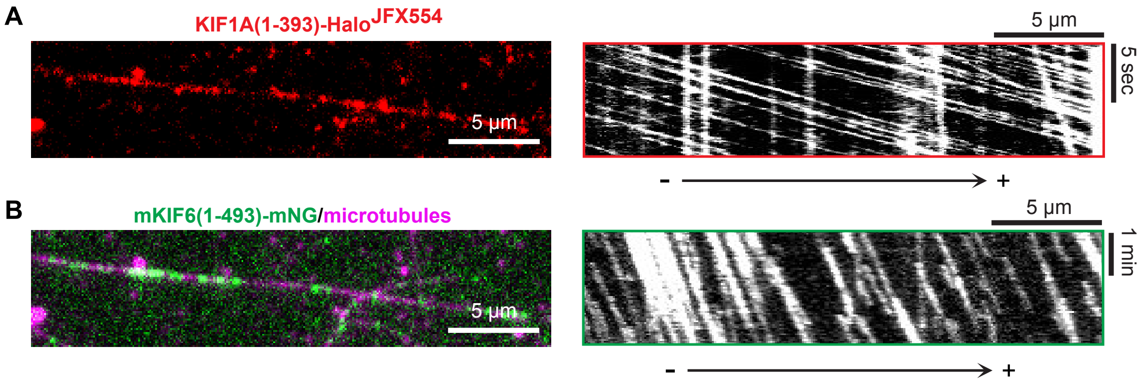

Fig. S2. mKif6(1-493)-mNG moved in the same direction as KIF1A, related to Fig. 1.

(A) Left, the representative still images from movies of KIF1A(1-393)-LZ-Halo^{JFX554} (red). Right, the representative kymograph of of KIF1A(1-393)-LZ-HaloJFX554. Time is on the y-axis (scale bar, 5 s) and distance is on the x-axis (scale bar, 5 μm).

(B) Left, mKif6 (1-493)-mNG (green) along taxol-stabilized microtubules (magenta). Right, the kymograph of mKif6 (1-493)-mNG at the same microtubules as shown in (A). Time is on the y-axis (scale bar, 5 s) and distance is on the x-axis (scale bar, 5 μm).

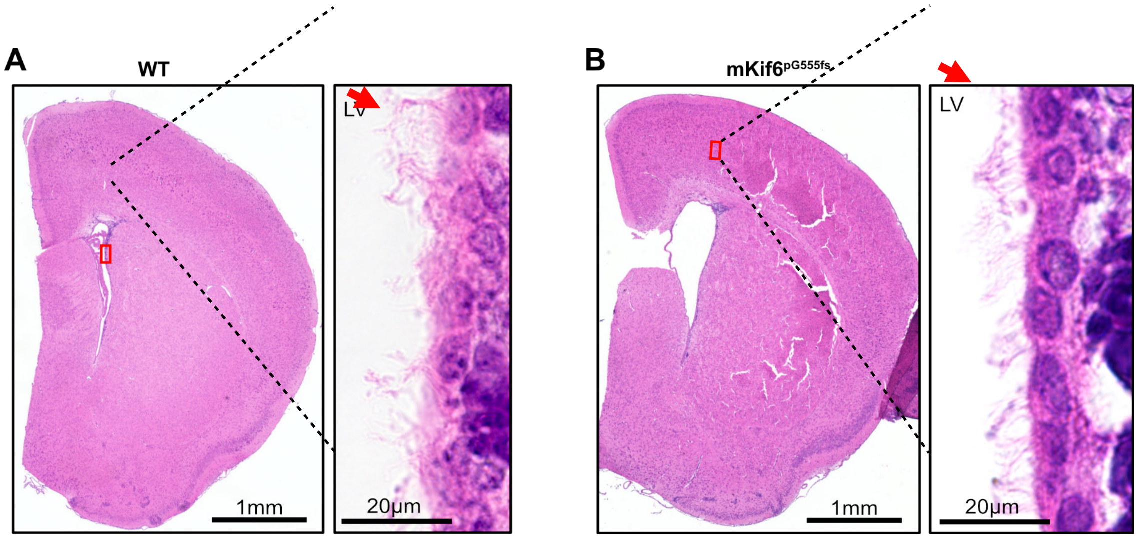

Fig. S3. Ependymal cells on LV wall in P14 WT or in *Kif6$^{p.G555fs}$* mice, related to Fig. 3.

(A, B) H&E-stained coronal section in WT (A) or *Kif6$^{p.G555fs}$* (B) mice brains at P14. Red boxes indicate the wall of Lateral ventricle (LV) expanded in the right panels. Arrows represent ependymal cilia.

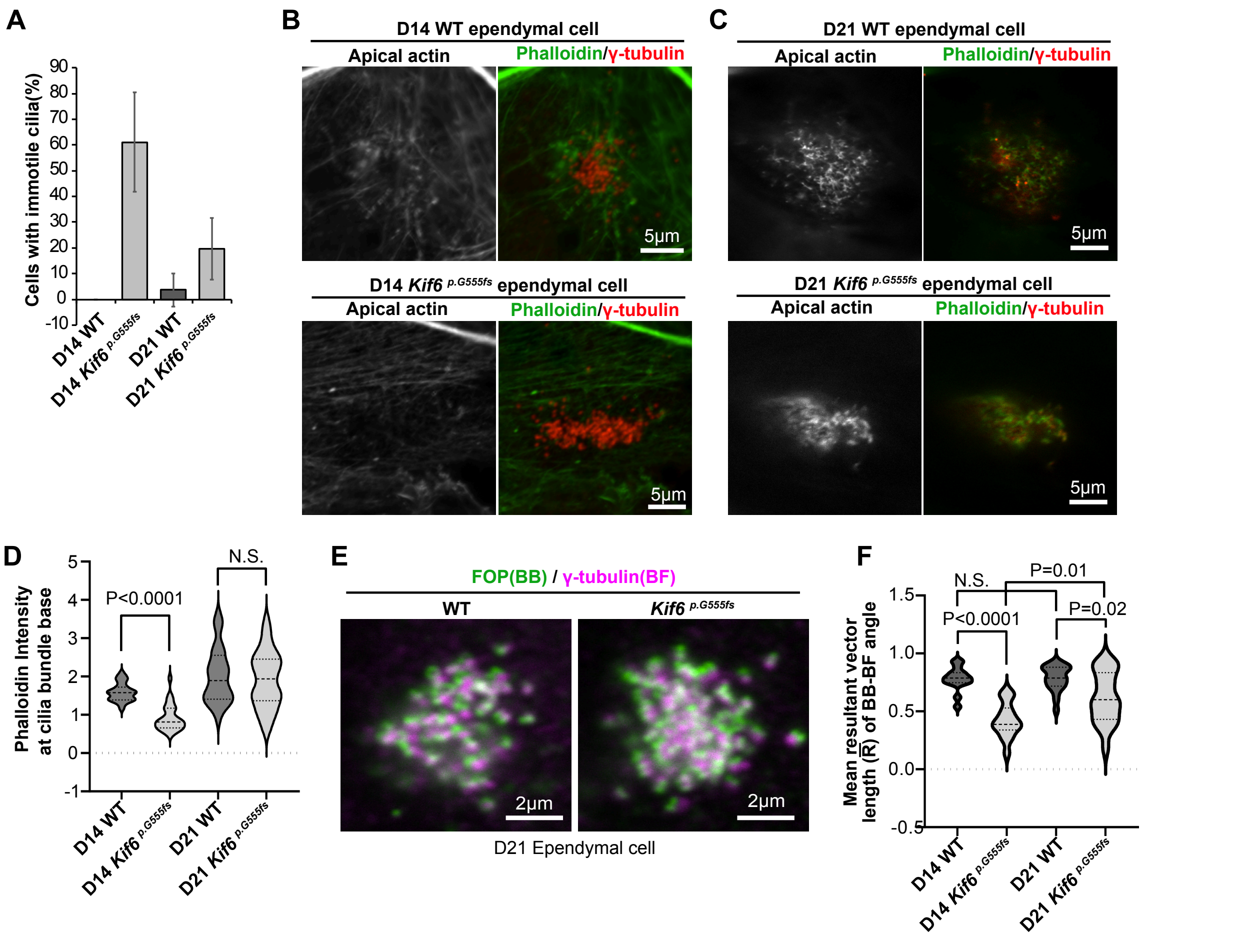

Fig. S4. Apical actin and rotational polarity in cultured ependymal cells, related to Fig. 3 and 6.

(A) Graph represents the percentage of cells with immotile cilia. The bar graph shows average and standard deviation of three different experiments in D14 WT (0%), D14 *Kif6$^{p.G555fs}$* (61.1% ± 19.2), D21 WT (3.7% ± 6.4) or D21 *Kif6$^{p.G555fs}$* (19.6% ± 12.0) ependymal cells.

(B) Representative images of immunostaining with Phalloidin (green) and γ-tubulin (red) in D14 WT (upper panels) or *Kif6$^{p.G555fs}$* (lower panels) ependymal cell.

(C) Representative images of immunostaining with Phalloidin (green) and γ-tubulin (red) in D21 WT (upper panels) or *Kif6$^{p.G555fs}$* (lower panels) ependymal cell.

(D) Graph represents Phalloidin intensity at cilia bundle base. The intensity of phalloidin at γ-tubulin dots area were divided by the intensity of Phalloidin at all area of apical cell cortex.

The violin plots show distribution with median and quartile in D14 WT (Median=1.579, n=27 cells from three different experiments), P14 *Kif6$^{p.G555fs}$*
(Median=0.811, n=27 cells from three different experiments), D21 WT
(Median=1.892, n=27 cells from three different experiments), or P14 *Kif6$^{p.G555fs}$*
(Median=1.939, n=27 cells from three different experiments). P-value was determined with the Mann Whitney test.

(E) Representative images of immunostaining with FOP (green) and γ-tubulin (magenta) in D21 WT (left panel) or Kif6p.G555fs (right panel) ependymal cell.

(F) Graph represents mean resultant vector length (R bar) of BB-BF angle that are measured in the immunostaining images as shown in Figure 5D and Figure S6D. The violin plots show distribution with median and quartile in D14 WT (Median=0.79, n=15 cells from two experiments), D14 *Kif6*$^{p.G555fs}$ (Median=0.39, n=15 cells from two experiments), D21 WT (Median=0.79, n=20 cells from three experiments), or D21 *Kif6*$^{p.G555fs}$ (Median=0.60, n=20 cells from three experiments). P-value was determined with the Mann Whitney test.

Disease Models & Mechanisms • Supplementary information

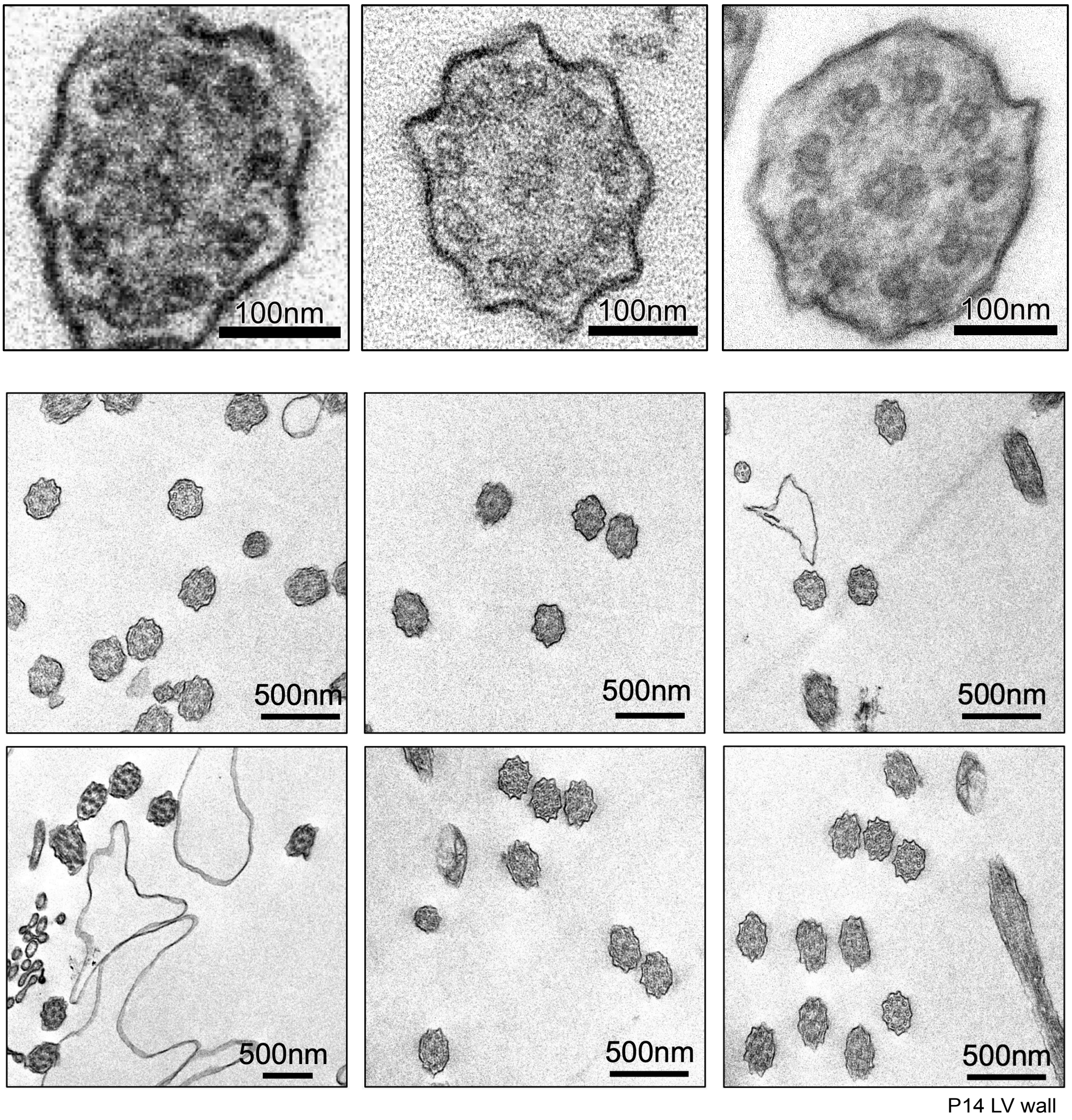

Fig. S5. TEM images in P14 *Kif6$^{p.G555fs}$* mice, related to Fig. 4

TEM images were taken from three different *Kif6$^{p.G555fs}$* mice LV walls at P14.

Disease Models & Mechanisms • Supplementary information

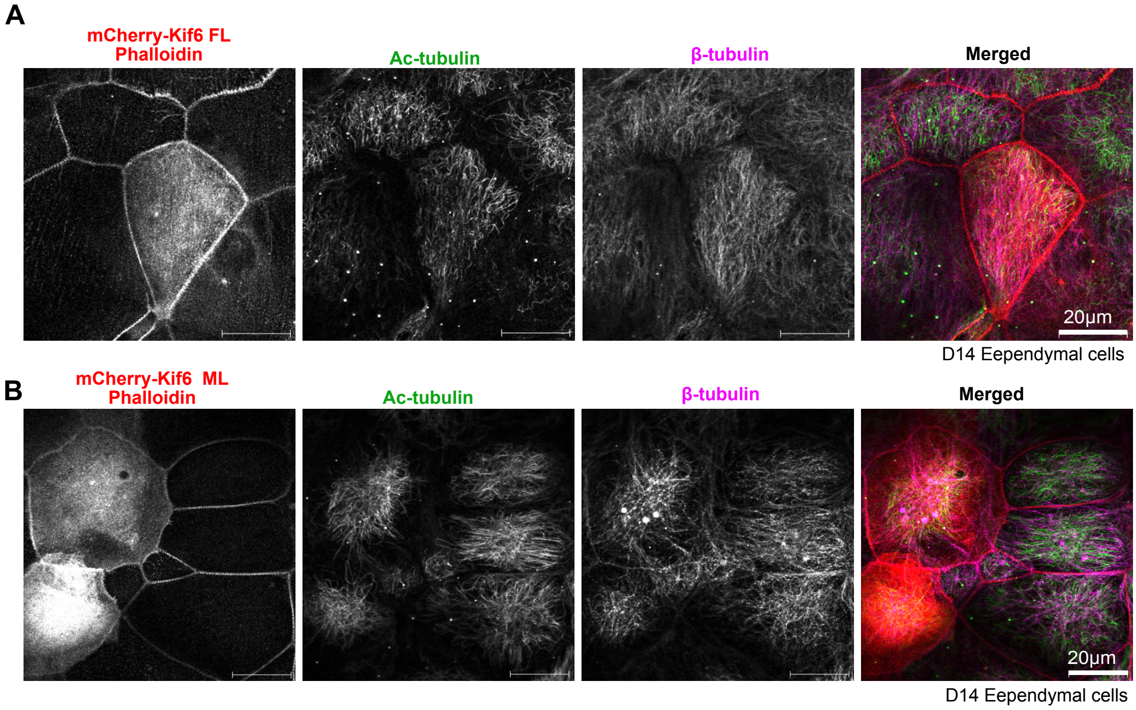

Fig. S6. Kif6 did not affect overall appearance of microtubules, related to Fig. 5.

(A, B) Representative images of immunostaining with Ac-tubulin (green) and β-tubulin (magenta) in mCherry-Kif6 FL (A) or mCherry-Kif6 ML (B) expressing D14 ependymal cell.

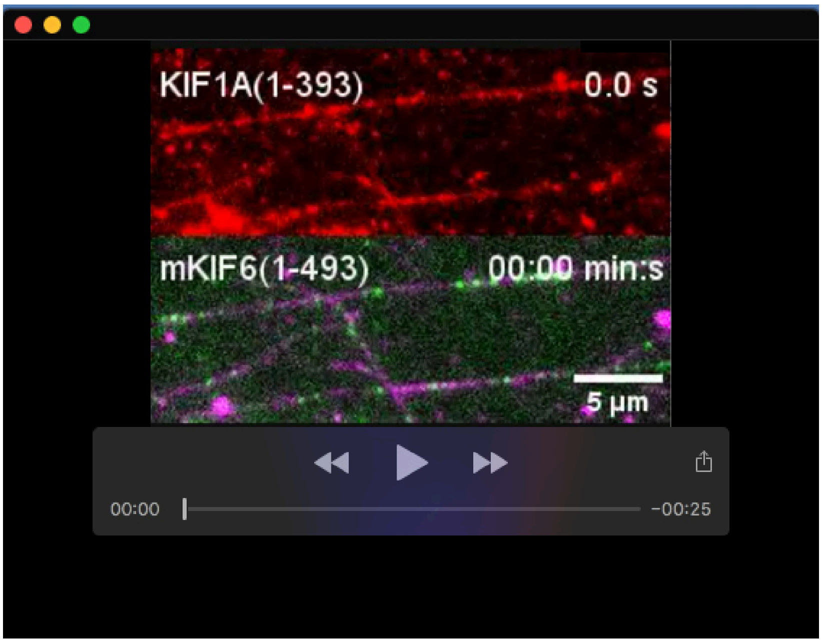

Movie 1. Movement of mKif6(1-493)-mNG and Kif1A (1-393)-LZ-HaloJFX554 7 along taxol-stabilized microtubules *in vitro*, shown in Fig. S3B.

Cell lysates from COS-7 cells expressing mKif6(1-493)-mNG (green) or KIF1A (1- 393)-LZ-HaloJFX554 (red) were added to a flow cell containing HiLy647-labeled taxol-stabilized microtubules (magenta). Images of KIF1A (1-393) were acquired at 1 frame every 100 ms for 61 frames and then images of Kif6(1-493) on the same microtubules were acquired at 1 frame every 3 s for 61 frames. The display rate of the movie is 30 fps.

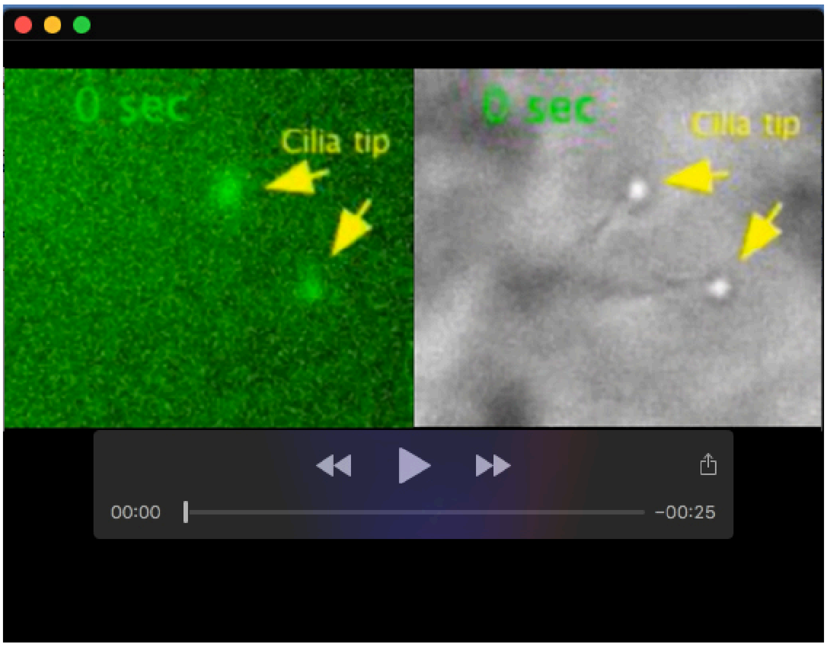

Movie 2. Live imaging of mKif6 (1-493)-mNG at a developing ependymal cilium, shown in Fig. 1G.

mKif6 (1-493)-mNG was transfected in D5 ependymal cell, and mKif6 (1-493)-mNG at short developing cilia was imaged at D7. Time-lapse imaging was acquired at 1 frame every 5s for 60 frames. The display rate of the movie is 3 fps.

Disease Models & Mechanisms • Supplementary information

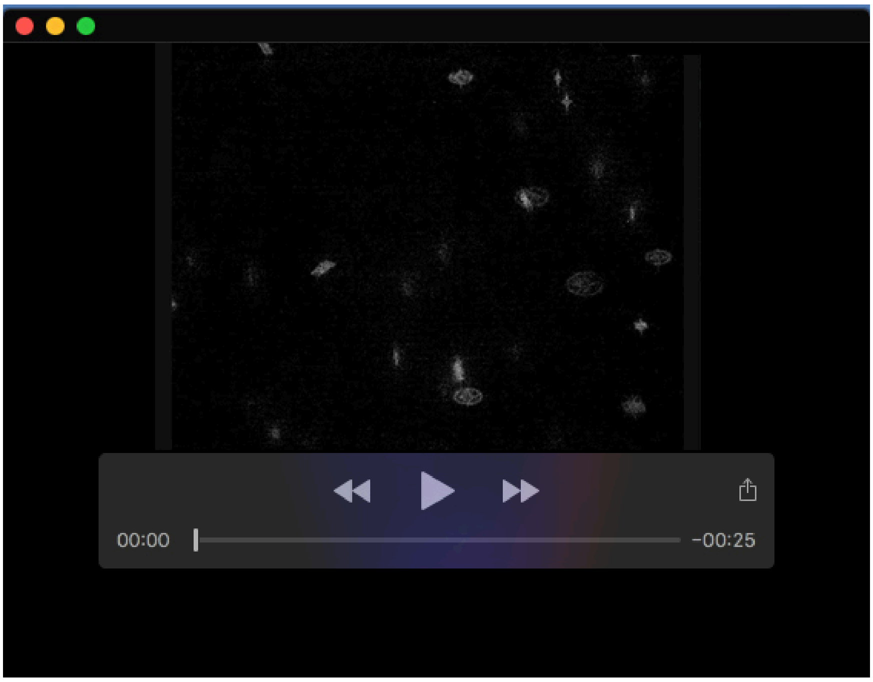

Movie 3. Beads migration on WT LVW, shown in Fig. 3E.

Fluorescent microsphere was placed on D12 WT LV wall. The beads migration was recorded in 10 fps for 10sec.

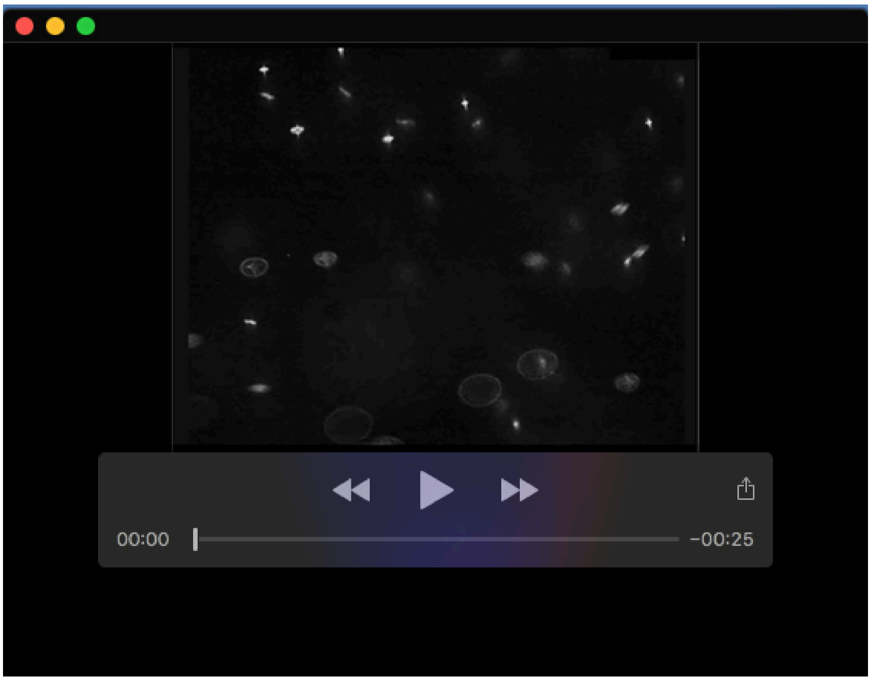

Movie 4. Beads migration on *Kif6* $^{p.G555fs}$ LVW, shown in Fig. 3E.

Fluorescent microsphere was placed on D12 *Kif6* $^{p.G555fs}$ LV wall. The beads migration was recorded in 10 fps for 10sec.

Disease Models & Mechanisms • Supplementary information

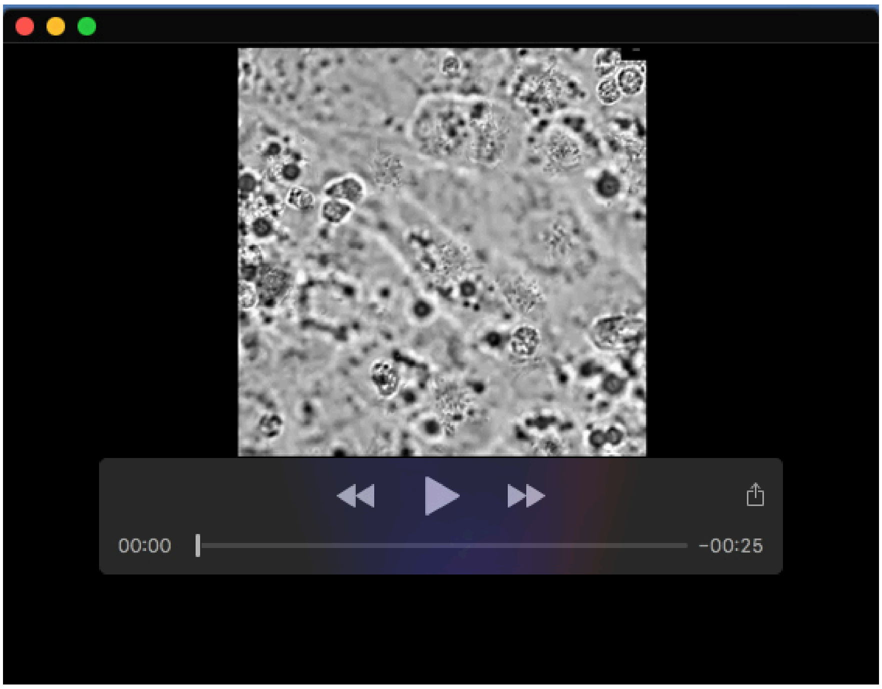

Movie 5. Live imaging of WT ependymal cell cilia at D14.

Primary cultured ependymal cells form WT LV walls were cultured on glass-bottom dish. Time-lapse imaging of DIC was acquired at 47 fps for 2 sec.

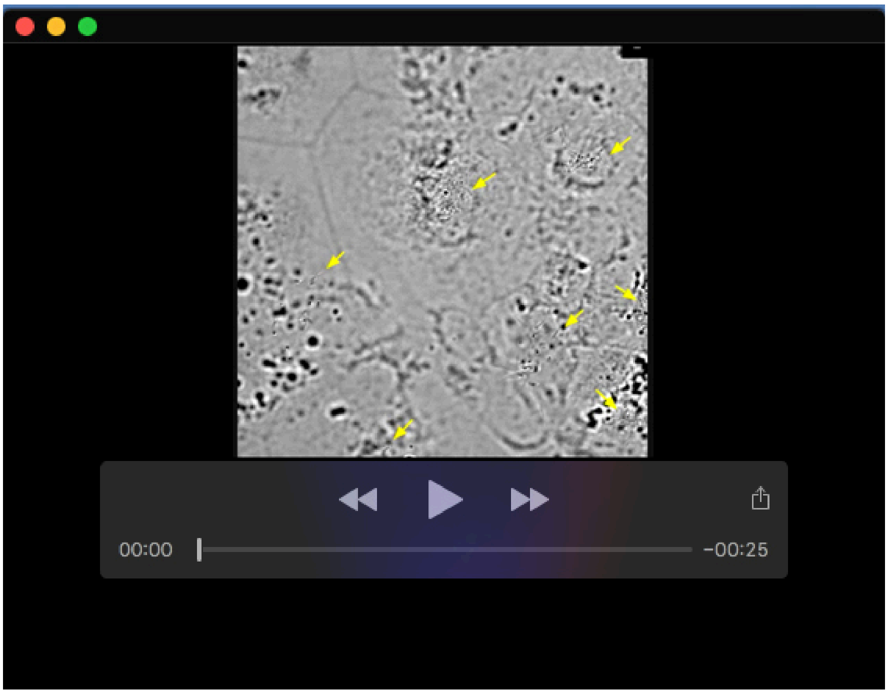

Movie 6. Live imaging of *Kif6* $^{p.G555fs}$ ependymal cell cilia at D14.

Primary cultured ependymal cells form *Kif6* $^{p.G555fs}$ LV walls were cultured on glass- bottom dish. Time-lapse imaging of DIC was acquired at 47 fps for 2 sec. Arrows indicate cilia on ependymal cells.

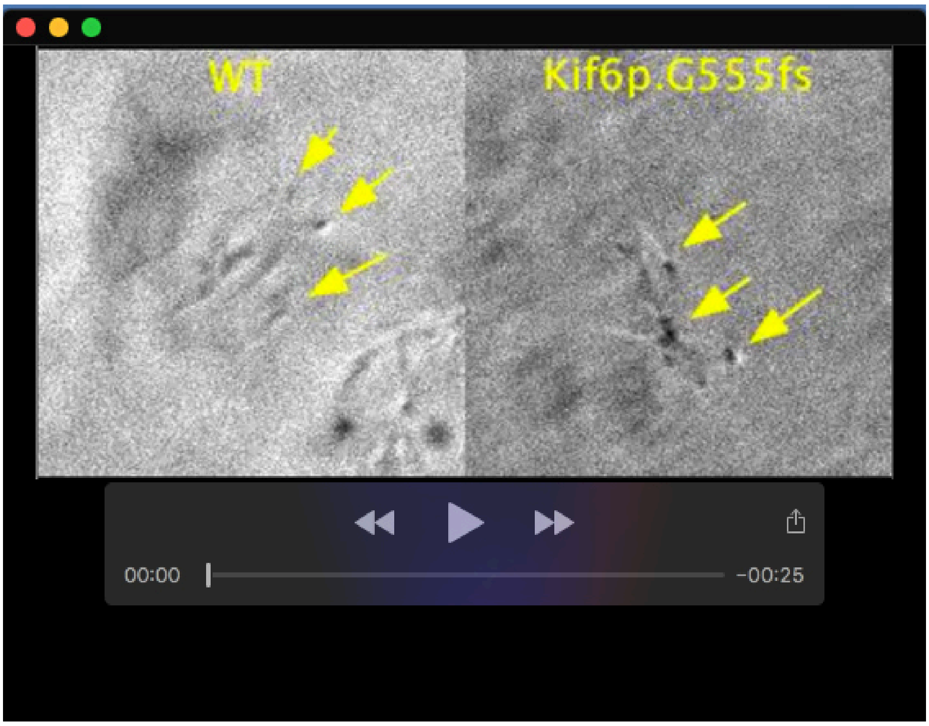

Movie 7. Live imaging of WT (left) and *Kif6* $^{p.G555fs}$

Primary cultured ependymal cells form WT or *Kif6* $^{p.G555fs}$ LV walls were cultured on glass-bottom dishes. Time-lapse imaging of DIC was acquired at 95 fps for 0.5 sec. Arrows indicate cilia on each ependymal cell.

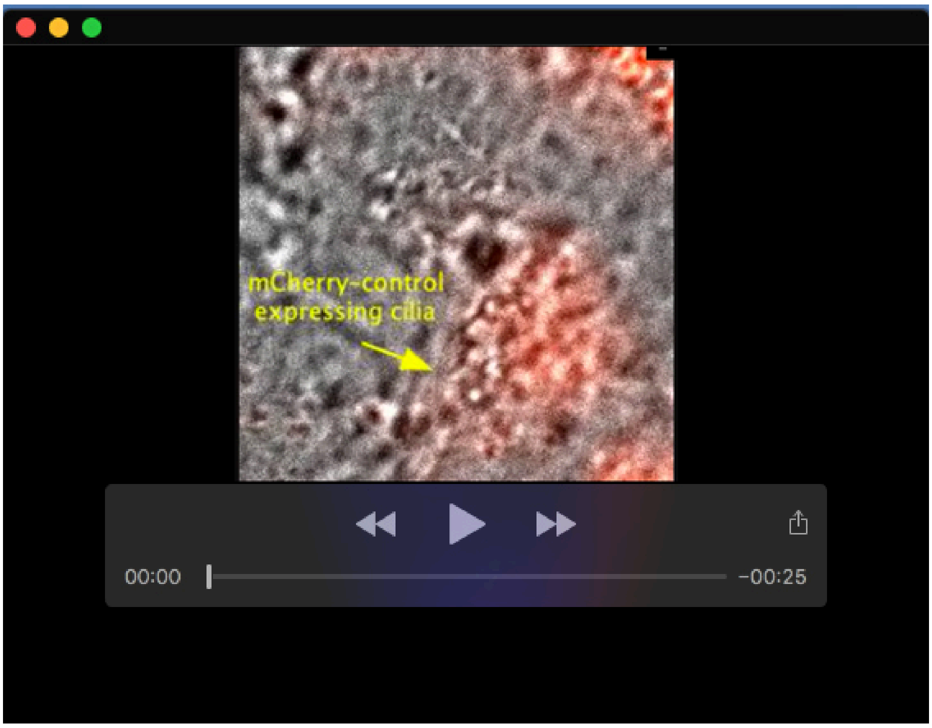

Movie 8. Live imaging of mCherry-expressing ependymal cell cilia at D14, related to Fig. 3J.

Primary cultured ependymal cells form WT LV walls were cultured on glass-bottom dishes and transfected with mCherry at D12. At D14, time-lapse imaging of DIC was acquired at 190 fps for 2 sec. mCherry signal with DIC image is shown in the first flame.

Disease Models & Mechanisms • Supplementary information

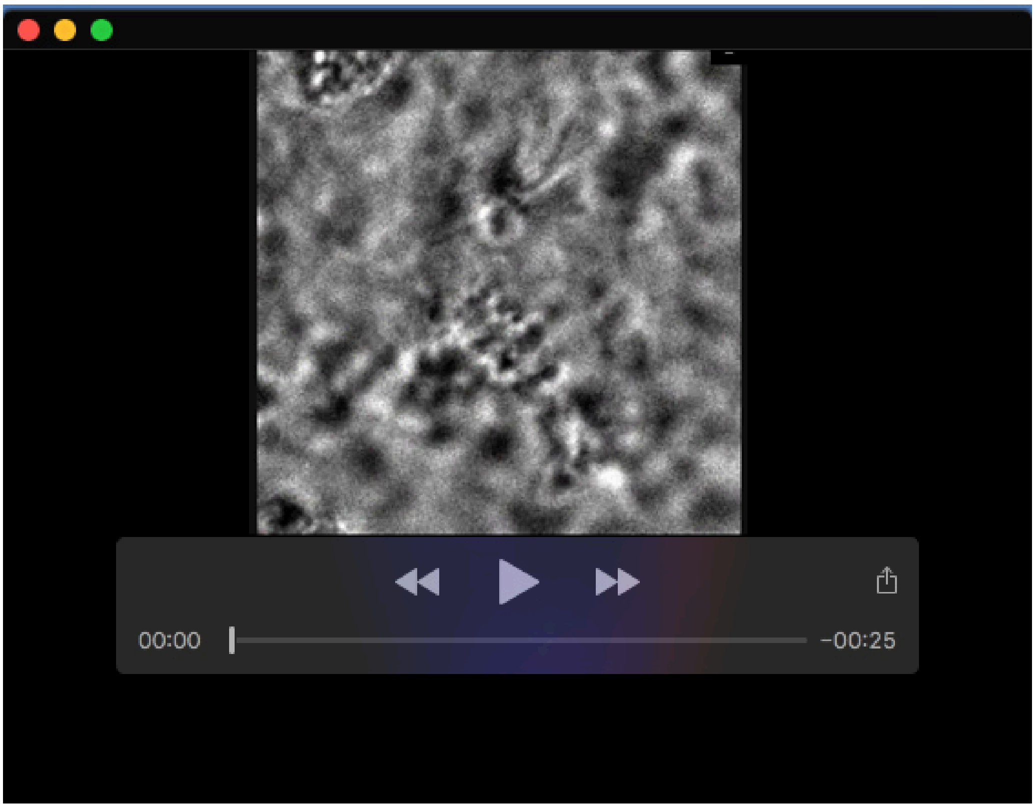

Movie 9. Live imaging of mCherry-Kif6 ML-expressing ependymal cell cilia at D14, related to Fig. 3J.

Primary cultured ependymal cells form WT LV walls were cultured on glass-bottom dishes and transfected with mCherry-Kif6 ML at D12. At D14, time-lapse imaging of DIC was acquired at 190 fps for 2 sec. mCherry signal with DIC image is shown in the first flame.
